# Multiple Mechanisms Promote the Retained Expression of Gene Duplicates in the Tetraploid Frog Xenopus laevis

**DOI:** 10.1371/journal.pgen.0020056

**Published:** 2006-04-28

**Authors:** Frédéric J. J Chain, Ben J Evans

**Affiliations:** Center for Environmental Genomics, Department of Biology, McMaster University, Hamilton, Ontario, Canada; Fred Hutchinson Cancer Research Center, United States of America

## Abstract

Gene duplication provides a window of opportunity for biological variants to persist under the protection of a co-expressed copy with similar or redundant function. Duplication catalyzes innovation (neofunctionalization), subfunction degeneration (subfunctionalization), and genetic buffering (redundancy), and the genetic survival of each paralog is triggered by mechanisms that add, compromise, or do not alter protein function. We tested the applicability of three types of mechanisms for promoting the retained expression of duplicated genes in 290 expressed paralogs of the tetraploid clawed frog, Xenopus laevis. Tests were based on explicit expectations concerning the *ka/ks* ratio, and the number and location of nonsynonymous substitutions after duplication. Functional constraints on the majority of paralogs are not significantly different from a singleton ortholog. However, we recover strong support that some of them have an asymmetric rate of nonsynonymous substitution: 6% match predictions of the neofunctionalization hypothesis in that (1) each paralog accumulated nonsynonymous substitutions at a significantly different rate and (2) the one that evolves faster has a higher *ka/ks* ratio than the other paralog and than a singleton ortholog. Fewer paralogs (3%) exhibit a complementary pattern of substitution at the protein level that is predicted by enhancement or degradation of different functional domains, and the remaining 13% have a higher average *ka/ks* ratio in both paralogs that is consistent with altered functional constraints, diversifying selection, or activity-reducing mutations after duplication. We estimate that these paralogs have been retained since they originated by genome duplication between 21 and 41 million years ago. Multiple mechanisms operate to promote the retained expression of duplicates in the same genome, in genes in the same functional class, over the same period of time following duplication, and sometimes in the same pair of paralogs. None of these paralogs are superfluous; degradation or enhancement of different protein subfunctions and neofunctionalization are plausible hypotheses for the retained expression of some of them. Evolution of most X. laevis paralogs, however, is consistent with retained expression via mechanisms that do not radically alter functional constraints, such as selection to preserve post-duplication stoichiometry or temporal, quantitative, or spatial subfunctionalization.

## Synopsis

Gene duplication plays a fundamental role in biological innovation but it is not clear how both copies of a duplicated gene manage to circumvent degradation by mutation if neither is unique. This study explores genetic mechanisms that could make each copy of a duplicate gene different, and therefore distinguishable and potentially preserved by natural selection. It is based on DNA sequences of the protein-coding region of 290 expressed duplicated genes in a frog, *Xenopus laevis,* that underwent complete duplication of its entire genome. Results provide evidence for multiple mechanisms acting within the same genome, within the same functional classes of genes, within the same period of time following duplication, and even on the same set of duplicated genes. Each copy of a duplicate gene may be subject to distinct evolutionary constraints, and this could be associated with degradation or enhancement of function. Functional constraints of most of these duplicates, however, are not substantially different from a single copy gene; their persistence in the first dozens of millions of years after duplication may more frequently be explained by mechanisms acting on their expression rather than their function.

## Introduction

By providing a redundant genetic template, gene duplication could relax purifying selection on one or both gene copies and facilitate functional divergence. Duplication catalyzes reproductive incompatibilities and speciation [1–3], facilitates exon shuffling [4] and microfunctionalization [5], buffers genetic pathways against null mutations [6], decreases pleiotropy [7], increases the diversity of gene expression [8,9], and increases specialization of genes and genetic pathways. Duplicated genes exchange information through recombination, gene conversion, and epigenetic processes [10]. However, unless natural selection favors the retained expression of both paralogs, mutations are generally expected to silence one gene copy soon after duplication [11,12]. Duplication by polyploidization, for example, is accompanied by extensive and rapid genome restructuring and gene silencing; gene silencing is achieved in a variety of ways including mutations in the protein-coding sequence or regulatory elements, and changes in methylation, histones, and chromatin structure [13–15]. In order to retain expression of both copies, evolutionary mechanisms must therefore counteract or exploit mutation-induced degeneration. Thus, the questions of how both paralogs retain expression, and how molecular evolution changes after duplication has captured the interest of evolutionary biologists.

**Figure 1:**
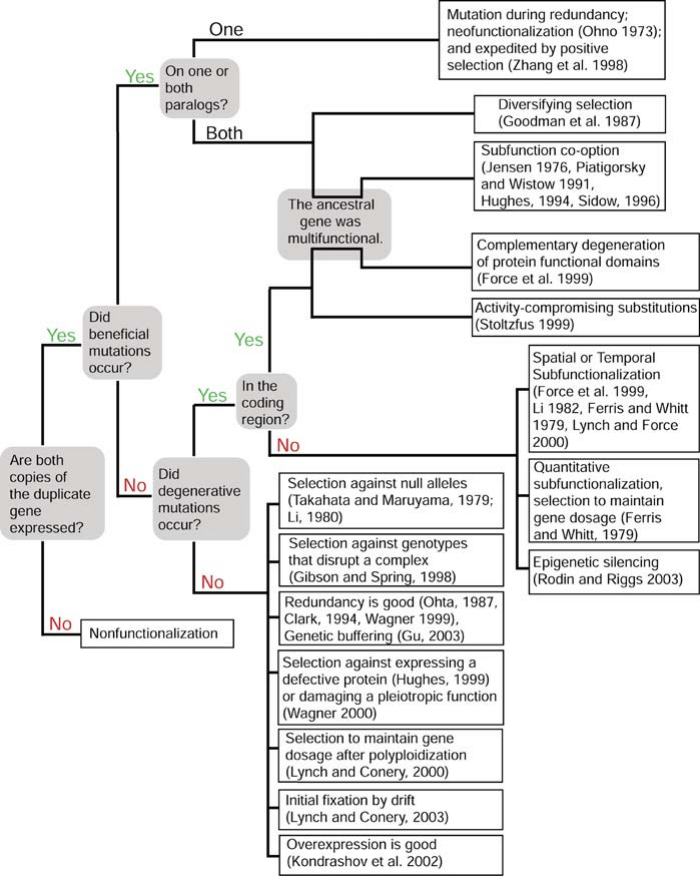
A Non-Exhaustive Diagram Relating Various Models for the Fate of Duplicate Genes Citations that either propose mechanisms or discuss them: Clark 1994 [100]; Ferris and Whitt 1979 [101]; Force et al. 1999 [17]; Gibson and Spring 1998 [74]; Goodman et al. 1987 [102]; Gu et al. 2003 [6]; Hughes 1994 [42]; Jensen 1976 [103]; Kondrashov et al. 2002 [19]; Li 1980 [104];Li et al. 1982 [105]; Lynch and Conery 2000 [1]; Lynch and Conery 2003 [106]; Lynch and Force 2000 [2]; Ohno 1973 [107]; Ohta 1987 [108]; Piatigorsky and Wistow 1991 [109]; Rodin and Riggs 2003 [110]).; Sidow 1996 [111]; Stoltzfus 1999 [60]; Takahata and Maruyama 1979 [112]; Wagner 1999 [53]; Wagner 2000 [113]; and Zhang et al. 1998 [68].

Central to our understanding of the fate of gene duplicates are the questions of whether paralogs evolve differently from singletons, whether they evolve differently from each other, and whether their retained expression is more frequently triggered by mechanisms that add, compromise, or do not alter protein function [1,16–20] (Figure 1). Molecular evolutionary analyses can be used to test the applicability of alternative explanations for the retained expression of duplicate genes that predict a unique molecular signature in the protein-coding portion in terms of the rates and locations of nonsynonymous substitutions, and the ratio of nonsynonymous substitutions per nonsynonymous site to synonymous substitutions per synonymous site (hereafter referred to as the *ka/ks* ratio).

Of course, these proposed mechanisms are not mutually exclusive, because they could operate concurrently, on different parts of the genes, inside and/or outside of the coding region, and at different times after duplication. Moreover, if these mechanisms involve positive selection on one or both paralogs, their genetic signature will be difficult to detect in old duplicates if positive selection occurred soon after duplication, on only a portion of amino acid sites, or if it was followed by a long period of purifying selection. Other obstacles to dissecting out these mechanisms include variation in the rate of evolution over time, between lineages [1,20], functional classes of genes [18], and genomic locations of each gene copy [21] and variation in the rate of gene duplication [22], saturation of synonymous substitutions [23], and mistaken identification of expressed duplicates that are actually pseudogenes or allelic variants. Nonetheless, if a particular mechanism operates for an extended period of time or on a large portion of the paralog(s), or if it involves a change in protein function or expression, it should be detectable by comparison to closely related orthologous singletons.

Clawed frogs (genera *Xenopus* and *Silurana*) offer a useful model system for exploring evolution of gene duplicates. Multiple species in this clade have undergone genome duplication via allopolyploidization and these polyploid genomes are primarily disomic in that each chromosome has only one homolog, as opposed to being polysomic, where multivalents form and recombination between paralogous loci is more prevalent [15,24–26]. Extant tetraploids originated once in *Xenopus* and once in *Silurana* [26], and as a result, duplicate genes originating from tetraploidization in *Xenopus* are the same age. Detailed studies have been performed on hundreds of expressed duplicate genes (Table S1), and synonymous substitutions are generally not saturated [27].

A landmark study by Hughes and Hughes [16] used this system to explore molecular evolution of 17 pairs of expressed gene duplicates in the tetraploid Xenopus laevis. They recovered evidence for an elevated *ka/ks* ratio after duplication, but still below the neutral expectation, and no evidence for a significantly different rate of nonsynonymous substitution relative to single-copy orthologs in mammalian outgroups. Their results are not consistent with neofunctionalization [28], wherein expression of duplicates is retained because one gene copy acquires novel function while the other carries out an ancestral function. Since this research was published, new mechanisms for duplicate gene retention have been proposed (Figure 1), and genomic sequences of the closely related diploid Silurana tropicalis have become available. In order to further evaluate these proposals, we have reanalyzed genes examined by Hughes and Hughes [16] and also deployed new data, for a total of 290 gene duplicates expressed in the tetraploid X. laevis (Table S1).

## Results

### The *ka/ks* Ratio, Expressed Paralogs in *X. laevis,* and Hypothesis Testing

Rates and types (nonsynonymous or synonymous) of substitution in the coding region are influenced by factors that are not directly linked to protein function, such as GC content, RNA secondary structure, and methylation [29–31], and also by factors that are related to protein activity, but not unique to a particular function, such as level of expression [32–35]. However, because nonsynonymous changes by definition affect the amino acid sequence of a protein, this class of substitution is more strongly affected by natural selection than synonymous substitutions. Evaluation of the *ka/ks* ratio therefore provides information on functional constraints on proteins, under the assumption that the effective population size does not change [36–39].

Unfortunately this assumption is rarely met. If the *ka/ks* ratio of low frequency polymorphisms is different from the *ka/ks* ratio of fixed differences, demographic changes will alter the *ka/ks* ratio of fixed differences by changing the fixation probability of polymorphisms. This is not a problem when comparing the *ka/ks* ratio (or the rate of nonsynonymous substitution) between paralogs in the same species because they share the same demographic history. However, unique demographic fluctuations could affect the *ka/ks* ratio of homologous genes in separate diploid and tetraploid species, even if selective constraints on proteins in these species were equal. For example, if mildly deleterious amino acid substitutions segregate at a low frequency, a reduction in population size of one species would increase the *ka/ks* ratio of fixed differences [37]. In comparing the *ka/ks* ratio of homologous genes in a diploid and a tetraploid species, we thus make the assumption that the effect of the unique demographic histories of each species is small compared to the effect of the unique selective constraints in these different types of genomes. In this study, we also do not have polymorphism information with which to distinguish fixed and segregating differences, and we therefore make a second assumption that the observed differences between paralogs are fixed.

**Figure 2:**
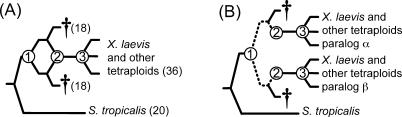
Putative Allopolyploid Evolution of the Tetraploid X. laevis Daggers indicate extinct diploid ancestors or genes. Nodes 1 and 2 correspond with the divergence and union, respectively, of two diploid genomes, and Node 3 marks the diversification of *Xenopus* tetraploids. (A) A reticulate phylogeny with ploidy in parentheses. (B) Nuclear genealogy assuming no recombination and no gene conversion between alleles at different paralogous loci (α and β). The dashed portion of the paralogous lineages evolved independently in different diploid ancestors.

We identified 290 paralogs expressed in X. laevis by searching the literature and molecular databases for sequences expressed at the RNA and/or protein level (Table S1). Tetraploidization of this species probably occurred via allopolyploidization (Figure 2). Both paralogs were used to identify an S. tropicalis ortholog (JGI, assembly 3.0). Phylogenetic and phenetic methods were used to confirm that these sequences were paralogous rather than allelic and that they originated from tetraploidization of X. laevis as opposed to a separate gene duplication event. By comparing each pair of paralogs to closely related orthologs from *S. tropicalis,* we minimize the confounding effects of functional differences in the comparison. Because genes in a polyploid are simultaneously duplicated, we have standardized across all duplicates the impact of variation in the genome-wide rate of evolution over time after duplication.

**Figure 3:**
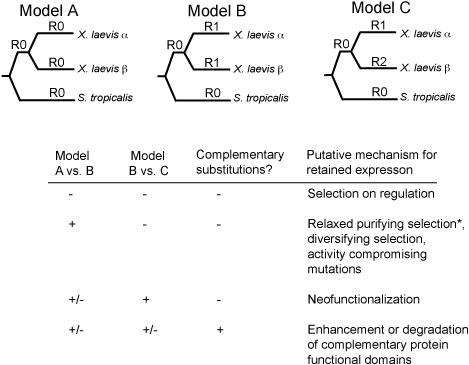
Assignment of Putative Retention Mechanisms Based on Molecular Changes in the Coding Region We assigned a retention mechanism to paralogs based on the results of three analyses. The first one compared a model with no change in the *ka/ks* ratio after duplication (Model A in which the *ka/ks* ratio on all branches is indicated by R0) to a model with a higher *ka/ks* ratio after duplication (Model B with *ka/ks* ratio R1 > R0). The second one compared a model with no difference in the nonsynonymous substitution rate (Model B, in which R0 and R1 are nonsynonymous rates on each branch) to a model with different rates of nonsynonymous substitution in each paralog (Model C in which R0, R1, and R2 are nonsynonymous rates on each branch), with the stipulation that the paralog with the higher nonsynonymous rate also have a higher *ka/ks* ratio than the slower paralog and a higher *ka/ks* ratio than the diploid lineage. The third analysis tested for complementarity of amino acid substitution in each paralog. In the table in the figure, a minus sign (−) indicates either no significant difference between the models or no significant complementarity of nonsynonymous substitutions. A plus sign (+) indicates a significant improvement in likelihood of the more parameterized model or significant complementarity of nonsynonymous substitution. An asterisk (*) denotes the caveat that an increased substitution ratio could stem from relaxed purifying selection and therefore be a consequence of rather than a cause for retention.

We assigned mechanisms for duplicate gene retention to each of these paralogs based on three analyses that test specific predictions about the *ka/ks* ratio and the rate and location of nonsynonymous substitutions in their coding region after duplication (Figure 3). Analysis 1 tests whether the average *ka/ks* ratio in both paralogs increased after duplication, and is consistent with diversifying selection, positive selection on a subset of sites, activity-reducing mutations, or relaxed purifying selection after duplication (which is probably a consequence rather than a cause of retained expression). Analysis 2 tests whether the nonsynonymous substitution rate differed between paralogs, and is consistent with neofunctionalization. Because variation in evolutionary rate due to genomic location could influence rates of nonsynonymous substitution, for the second test we imposed the criterion that the *ka/ks* ratio of the paralog with the significantly higher rate of nonsynonymous substitution be higher than the *ka/ks* ratio of the other paralog and also higher than the *ka/ks* ratio of the singleton lineage, but we do not stipulate that the higher ratio be significantly higher. Analysis 3 tests whether the pattern of substitution in each paralog was complementary in that substitutions occurred in different parts of each paralog. This pattern is consistent with either complementary degeneration or enhancement of different protein functional domains. For each gene, we applied the sequential Bonferroni correction for these three tests [40].

### Diversifying Selection, Activity-Reducing Substitutions, and/or Relaxed Purifying Selection in Both Paralogs

We compared the likelihood of a model with a higher *ka/ks* ratio after duplication (Model B in Figure 3) to a model with no change in the *ka/ks* ratio (Model A in Figure 3). Thirty-eight out of 290 of these paralogs (13%) have a significantly higher average *ka/ks* ratio than the diploid lineage (even though this ratio does not exceed neutral expectations), but based on other tests, they have a similar rate of nonsynonymous substitution between paralogs and do not have a complementary pattern of nonsynonymous substitution. This difference is significant table-wide (Fisher's test; *p <*< 0.0001).

Interestingly, the diploid lineages of the alpha and beta globin genes acquired nonsynonymous substitutions much faster than their paralogous lineages and also much faster than other genes (Tables S2 and S3). The *ka/ks* ratios over all sites of the diploid alpha and beta globin are near neutral expectations (0.799 and 1.068, respectively; Table S2).

### Neofunctionalization

Under the neofunctionalization hypothesis, one paralog carries out the ancestral (pre-duplication) function and the other paralog acquires a useful novel function due to amino acid changes during a period of relaxed purifying selection. A prediction of neofunctionalization is that one paralog acquires nonsynonymous substitutions at a different rate than the other paralog and also faster than a homologous singleton. We tested a neofunctionalization model that has a different rate of nonsynonymous substitution on each paralog (Model C in Figure 3). This was compared to a null model with an equal rate of nonsynonymous substitution in each paralog (Model B in Figure 3). With the criterion that the faster paralog also have the highest *ka/ks* ratio, an individually significant difference in nonsynonymous rates was achieved for 40 genes (Table S3) and this difference is significant table-wide (Fisher's test; *p =* 0.0004). This significant difference between nonsynonymous but not synonymous substitutions was also confirmed with an alternative statistical framework (see below). After correcting for multiple tests, 18 out of 290 of these paralogs (6%) are individually consistent with the neofunctionalization model and also do not have a complementary pattern of substitution.

**Figure 4:**
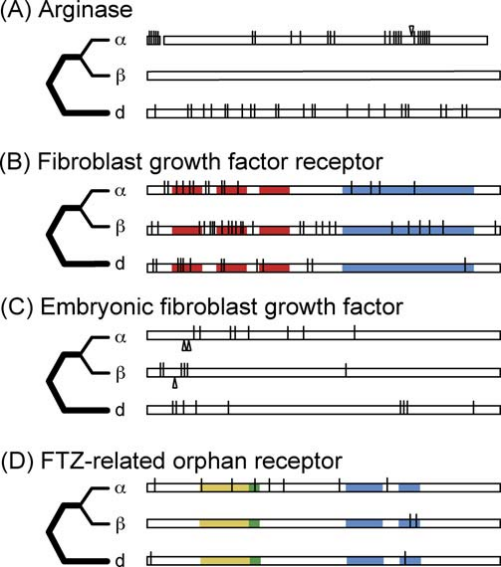
Nonsynonymous Substitutions in Each X. laevis Paralog (α and β) and the Diploid Lineage in Representative Genes Substitutions in the diploid lineage (d) occurred on the thick branches in the rooted topologies to the left of each locus. (A) *liver-type arginase,* (B) *fibroblast growth factor receptor* (FGFR), (C) *embryonic fibroblast growth factor* (EFGF), and (D) *FTZ-F1–related orphan receptor*. In (A) a gap indicates a single amino acid deletion, an arrow above the paralog indicates a single amino acid insertion, and this paralog is shortened due to an early stop codon. In (B) three red boxes and a blue box indicate three immunoglobulin domains and a tyrosine kinase domain. In (C) arrows below the paralog indicate predicted cleavage sites in each paralog [79]. In (D) yellow, green, and two lighter blue boxes indicate the DNA-binding C-domain, FTZ-F1 box, and DNA binding domain regions II and III [80].

An extreme scenario of neofunctionalization would involve one paralog remaining unchanged after duplication and the other paralog acquiring many substitutions. Interestingly, X. laevis paralogs of liver-type arginase have this genetic signature (Figure 4A). One paralog (X69820) incurred an in-frame deletion of one amino acid, an in-frame insertion of one amino acid, a new stop codon that terminates the protein seven codons upstream from the other paralog, and 25 amino acid substitutions. The other paralog (BC043635) is identical to the maximum likelihood reconstruction of the ancestral sequence, although a maximum parsimony reconstruction of this ancestral sequence suggests that two synonymous substitutions occurred in this paralog. This paralog (BC043635) is similar in size to S. tropicalis and to outgroups such as humans and mice, indicating that the indels occurred in the other paralog (X69820). Of course, this pattern of substitution could also occur if the rapidly evolving paralog were a pseudogene. However in the case of liver-type arginase, polyclonal antibodies generated from protein translated from cDNA of the rapidly evolving paralog recognize two differently sized proteins in tadpole livers, but only one size in adult liver [41]. Although this would suggest expression of both paralogs at the protein level, cross hybridization to other genes or splice variants is a concern, and further studies are needed to confirm expression and translation of both of these paralogs.

### Complementary Substitutions

Another way that the retained expression of paralogs could be promoted is if different functional domains on each paralog were enhanced or degraded [17,42]. These mechanisms predict a complementary pattern of substitution on each paralog, and this pattern is not expected if neofunctionalization or regulatory changes drive their retained expression. We tested this possibility with the paralog heterogeneity test [43] and the runs test for dichotomous variables [44], after excluding genes with two or fewer substitutions in one or both paralogs. Using a more conservative null distribution than Dermitzakis and Clark [43], the paralog heterogeneity test identified more clustered nonsynonymous substitutions than expected by chance, depending on the number of domains assumed (235 genes tested, 13 or 18 genes were significant under the assumption of two or three domains at *p <* 0.05, Table S4). The runs test identified more genes with runs of nonsynonymous mutations on the same paralog than expected by chance (235 tests, 19 significant at *p <* 0.05, Table S4). Some duplicates were identified by both tests, and a few of these genes appear to have complementary substitutions in positions that correspond to distinct functional domains (See below). We used the lowest *p*-value from both methods for Bonferroni correction across these analyses (Figure 3).

As a qualitative test for Type I error, we also performed these tests on synonymous substitutions because we would not expect this class of mutations to be more heterogeneous in duplicates than in singletons. When synonymous substitutions were analyzed, both tests identified more significantly complementary mutations than expected by chance. The paralog test identified 24 or 22 out of 286 genes tested under the assumption of two or three domains, and the runs test identified 22 genes (*p <* 0.05; Table S4). One explanation for this observation is that synonymous substitutions of some paralogs are complementary. Synonymous substitutions can, for example, be heterogeneous [45]. Another explanation is that, although these tests help target some candidates for retention by subfunction co-option or subfunction partition in the coding region, both may suffer from Type I error. In any case, tests for complementary nonsynonymous and synonymous substitutions are both significant table-wide (*p =* 0.001 and *p <* 0.0001, respectively).

According to these tests, eight out of 235 of these paralogs (~3%) exhibit a significant complementary pattern of nonsynonymous substitution. One of them, *fibroblast growth factor receptor* (FGFR), also had a significantly different rate of nonsynonymous substitution. This could be explained by differently sized functional domains being co-opted or degraded, or by a different number of domains being altered in each paralog. In FGFR, it is a combination of these possibilities (Figure 4B). This gene has three immunoglobulin domains that are roughly 70 amino acids long, and one tyrosine kinase domain that is roughly 300 amino acids long. Four out of five substitutions in the first immunoglobulin domain are in one paralog (M55163) whereas the second immunoglobulin domain has five out of seven unique mutations in the other paralog (U24491) plus one in the same position in both paralogs. Six out of six substitutions in an approximately 115 amino acid long region between the third immunoglobulin domain and the tyrosine kinase domain are in one paralog (U24491). The tyrosine kinase domain has a similar number of mutations in both paralogs (four or five), but their distribution differs in that each paralog has most of its substitutions in either the beginning or the end of this domain.

### Tests over Multiple Loci: Codon Bias, Evolutionary Rates, and Functional Categories

Codon bias affects the *ka/ks* ratio due to selection on synonymous sites, and this bias could change after gene duplication, especially if it is linked to expression levels. However, we did not find a significant partial correlation between codon bias and the number of extra copies (zero or one) of the gene over all loci when the effect of the number of synonymous substitutions is held constant (*r* = −0.0004, *t_s_* = 0.0068, *df* = 287, *p* = 0.4973) or over just the loci with a significantly higher average *ka/ks* ratio (*r* = −0.0470, *t_s_* = 0.3908, *df* = 68, *p* = 0.3481). This indicates that the elevated *ka/ks* ratio after duplication in some paralogs cannot be attributed to increased selection on synonymous sites after duplication.

To further explore the null hypothesis of equal evolutionary rates in each paralog, we developed a method to use the equal mean Skellam distribution framework proposed by [46] over multiple loci. The null hypothesis of this test is that the number of nonsynonymous substitutions on each paralog follows the same Poisson distribution (i.e., the paralogs have equal rates). We used permutations to derive a probability distribution for the difference in the number of substitutions observed between all paralogs, and performed simulations to evaluate whether the observed distribution was significantly different from the expected equal mean Skellam distribution. To minimize the impact of variation in evolutionary rate due to genomic location, we restricted our analysis to genes in which synonymous substitutions met Poisson expectations (i.e., that the mean number of substitutions equal the variance in the number of substitutions); 260 out of the 290 genes met this criterion (89%). As a conservative measure, we also excluded one gene *(met mesencephalon-olfactory transcription factor 1)* from this analysis because we suspect a sequencing error increased the number of nonsynonymous substitutions of one paralog (AF041138), causing a run of eight amino acid differences that could be eliminated by shifting the nucleotide alignment out of frame by one base pair.

**Figure 5:**
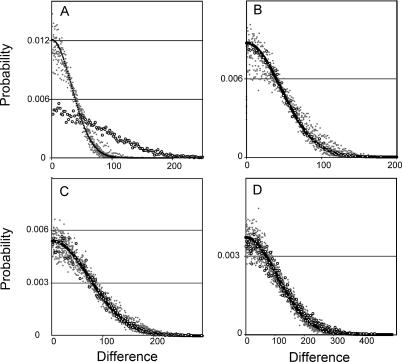
Probability Distribution of the Difference in the Number of Substitutions in Concatenated Paralogs (“Superparalogs”) Analysis was performed on concatenated data from (A) nonsynonymous substitutions of paralogs identified by the likelihood analysis as having asymmetric rates of evolution, (B) synonymous substitutions of these paralogs, (C) nonsynonymous substitutions of the other paralogs that were not identified as having asymmetric rates and (D) synonymous substitutions of these paralogs. Black circles are the expected Skellam distributions, gray dots are d_SP_ distributions from ten example simulations (out of 1,000 total), and white circles are the observed distribution of superparalog differences.

This analysis confirms the results of the likelihood test for unequal rates of nonsynonymous substitution (Analysis 2). The set of genes with an individually significant difference in nonsynonymous rates according to Analysis 2 also have a significant departure from the equal mean Skellam distribution null hypothesis for nonsynonymous substitutions (36 genes were analyzed; λ_ML_ = 543, *p <* 0.001, Figure 5A) even though, as expected, synonymous substitutions of these genes were not significantly different (λ_ML_ = 1039, *p =* 0.857, Figure 5B). The other genes did not have a significant departure from the equal means Skellam distribution null hypothesis for nonsynonymous (224 genes were analyzed; λ_ML_ = 2,737, *p =* 0.505, Figure 5C) or synonymous substitutions (λ_ML_ = 5,760, *p =* 0.124, Figure 5D). Thus, even after excluding loci with synonymous substitutions that do not meet Poisson expectations and also a locus with a potential sequencing error, these results strongly reject the null hypothesis of equal evolutionary rates in about 14% of these genes. The estimated percentage of genes consistent with neofunctionalization (6%) is lower because it is calculated in the context of multiple tests on each gene.

We also explored whether expression of paralogs of certain functional categories tends to be retained by a particular type of mechanism. Second-level gene ontology annotations from the three main categories (Biological Process, Molecular Function, and Cellular Component) were assigned to X. laevis paralogs based on the annotations (when available) of the most homologous hits that were obtained with the Gene Ontology Consortium Browser and BLAST tool (http://www.godatabase.org). After correcting for multiple tests, we did not recover a significant overrepresentation of retention mechanisms in any of the functional classes based on a hypergeometric distribution performed with GeneMerge [47]. In contrast, we find that expression of paralogs within functional classes are consistent with a diversity of mechanisms.

### Selective Constraints of Most Paralogs Are Not Significantly Different from an Orthologous Singleton

In gene duplication by polyploidy, as opposed to by doubling of a single gene or a fragment of the genome, selection to maintain protein stoichiometry could play a prominent role in preserving both copies of a duplicate gene because entire genetic networks are duplicated. In a polyploid genome, spatial, quantitative, or temporal subfunctionalization of expression could also promote retained expression of duplicate genes. Under these hypotheses, functional constraints (and pleiotropic interactions) of both paralogs are similar and nonsynonymous substitutions would not be in complementary locations in each paralog because each one performs an identical function to their singleton ancestral gene (though perhaps within a marginalized expression domain).

In all paralogs the average *ka/ks* ratio over all sites is less than one, indicating that the impact of purifying selection after duplication is pervasive (Table S2). However, in 226 out of 290 genes (78%), the average *ka/ks* ratio was not significantly higher after duplication, neither paralog had a significantly higher rate of nonsynonymous substitution and higher *ka/ks* ratio than the orthologous diploid lineage, and there was not a significantly complementary pattern of nonsynonymous substitution in each paralog. The degree to which this estimate is inflated by Type II error is expected to vary from gene to gene depending on the power of each test, the amount of data, unique parameter values of the data (transition/transversion ratios, base frequencies, branch lengths), and the degree to which the data depart from the null hypothesis.

### Age of *Xenopus* Paralogs

If tetraploidization occurred by allopolyploidization, paralogs of X. laevis co-evolved in the same genome for a period of time that is shorter than the duration of their divergence (Figure 2). Using a relaxed molecular clock calibrated with geological and fossil data, we estimated the divergence time of *Xenopus* paralogs based on portions of the *RAG1* and the *cytokine receptor 4* genes. To avoid the possibility that an accelerated rate of nonsynonymous substitution after duplication could affect our estimates, we included only synonymous substitutions at fixed amino acid positions and four-fold degenerate sites. This analysis indicates that divergence of *Silurana* and *Xenopus* occurred 53 million years ago (mya) with a 95% confidence interval (CI) of 40–80 mya. The age of the most recent common ancestor of the α and β paralogs (Node 1 in Figure 2), which corresponds to the diversification of the diploid ancestors of *Xenopus* tetraploids, is estimated to be 41 mya (CI: 29–66 mya). Diversification of *Xenopus* tetraploids (Node 3 in Figure 2) is estimated to be about 21 mya (CI: 13–38 mya). We did not directly estimate the timing of allopolyploidization (Node 2 in Figure 2) because no extant descendant of the most recent diploid ancestor of X. laevis is known [26]. Thus we have narrowed down the age of *Xenopus* genome duplication to between 21 and 41 mya, but with broad confidence limits for these upper and lower boundaries.

The estimated time of divergence of *Silurana* and *Xenopus* (~53 mya) and the estimated time of tetraploid divergence (~21 mya) are less than the corresponding estimates based on mitochondrial DNA (~64 mya and ~42 mya, respectively) [48]. However, all of them are about twice as old as estimates based on immunological distances of antiserum to albumin (about 30 and 10 mya, respectively) [49]. We suspect that these immunological distances could underestimate divergence between sister tetraploid species because intraspecific divergence between expressed paralogs is similar to or greater than interspecific divergence between paralogs (Figure 2). Divergence between expressed paralogs in a tetraploid could also reduce immunological distances between a tetraploid species and a diploid species as compared to two similarly diverged diploid species. Another estimate of 110 million years for the divergence of *Silurana* and *Xenopus* [50] is clearly an overestimate because it is based on globin proteins with an atypically rapid rate of evolution in diploid clawed frogs (Table S2).

## Discussion

Genome duplication provides an approximation of the assumption of initial redundancy made by some models for retained expression of gene duplicates [51–53], because intact regulatory elements are duplicated with the protein-coding region. However, many duplicates in diploid genomes are gene fragments or have incomplete regulatory elements [54], and extensive and rapid genome restructuring can also fragment protein-coding and regulatory regions in polyploids [13,55]. Initial population genetic dynamics of duplicates in polyploid genomes differ from those of duplicates in diploid genomes. In a diploid genome duplicates must become fixed, whereas in a polyploid genome duplicates must stay fixed. Selective pressures to maintain expression stoichiometry also differ in each system; duplication by polyploidy does not change stoichiometry, but singleton duplication does [1]. Nonetheless, a recent comparison of expressed duplicates derived from whole genome duplication to paralogs from smaller-scale duplication found that although the functional attributes differ between these types of expressed duplicates, molecular evolutionary changes are analogous [56].

Our results suggest that most of these paralogs do not have significantly different selective constraints from a diploid ortholog. The extent to which this applies to duplicate genes in diploid species depends how many of these X. laevis paralogs are expressed as a result of attributes that are unique to polyploids (such as selection to maintain the stoichiometry of expression in a duplicated genome) versus other mechanisms common to both types of genomes (such as quantitative, spatial, and regulatory subfunctionalization). Retained expression of duplicates in either type of genome might be favored, for example, if overexpression is advantageous [19].

### Increased *ka/ks* Ratio after Duplication

Other studies have reported a higher *ka/ks* ratio following duplication, and the magnitude that this ratio increases differs among groups [1,16,19,20,42,57,58], but see [18]. Conservative sites are more apt to change after duplication [59], and a burst of nonsynonymous substitutions following duplication is suggested by comparison of young to old duplicates [20]. This change is often attributed to relaxed purifying selection following duplication, but could also occur if some aspects of ancestral function disappear in both copies after duplication. The ability to self-dimerize, for example, is lost when a duplicated homodimer becomes a heterodimer. An increased tolerance of activity-reducing mutations in both paralogs could also occur such that the function of both is needed to recover the activity of the singleton ancestor [60].

**Figure 6:**
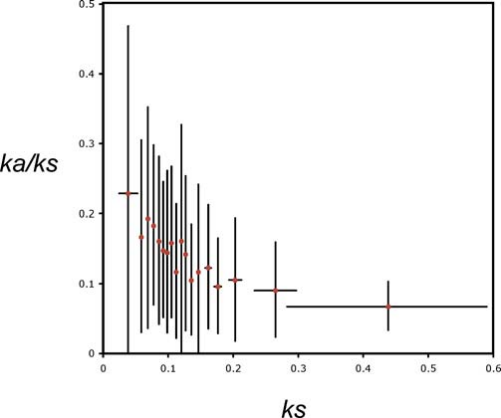
The Observed Relationship between *ka/ks* and *ks* The observed relationship between *ka/ks* and *ks* corresponds with simulations that predict a negative relationship under neutral or near-neutral evolution of synonymous substitutions because of stochastic sampling of synonymous substitutions at in slowly evolving or young genes [61]. The plot shows the average *ka/ks* ratio on each branch of 290 genealogies versus average *ks* of bins of 50 lineages ranked by *ks* of each one. The last bin has only 20 lineages. Bars indicate the standard deviation of each bin.

Interestingly, age discrepancies between the duplicates and the singletons could affect comparison of the *ka/ks* ratio [20]. An unexpected positive correlation between *ka/ks* and *ks* was reported in comparisons between distantly related orthologs of some mammals, but a negative correlation exists between closely related mammalian comparisons [61]. In the closely related sequences, a negative correlation is expected as a result of stochastic sampling of synonymous substitutions at low mutation rates [61]. Consistent with this expectation, linear regression of data from clawed frogs indicates a weak negative correlation between *ka/ks* and *ks* (*r^2^* = 0.055, unpublished data); this relationship is more obvious when data are binned (Figure 6).

**Figure 7:**
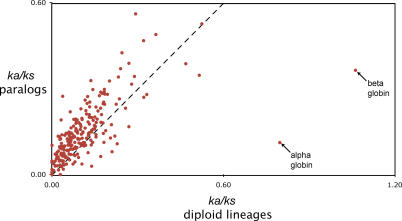
The *ka/ks* Ratio of Genes with No Significant Difference before and after Tetraploidization The *ka/ks* ratio is often slightly higher in the paralogs (above the dashed line), even though this average is not significantly higher than the diploid lineage. Only ratios from genes with no significant difference are shown (226 out of 292 genes). A dashed line indicates an equal *ka/ks* ratio before and after duplication.

The duration of divergence of X. laevis paralogs is twice their age, or about 82 million years. We estimate that the total divergence time of the diploid lineage (between node 1 and S. tropicalis in Figure 3) is about 75 million years. Because the ages of these lineages are similar, we expect that the effect of stochastic sampling of synonymous substitutions would also be similar [61]. However, some paralogs have a significantly higher *ka/ks* ratio, and many of them have a slightly higher *ka/ks* ratio after duplication even though the difference is not significant (Figure 7). Thus, these data provide strong evidence that some duplicates (~13%) evolve differently, averaged over both paralogs, than singletons, even though a significant change was not observed in the majority of these loci.

### Asymmetric Evolutionary Rates

The neofunctionalization hypothesis for the retained expression of duplicate genes has been criticized because expression of duplicate genes is retained more frequently and for a longer time than expected if this were the principal mechanism for retention [59,62–65]. This hypothesis also lacks a known mechanism for sequestering beneficial mutations to only one of the two duplicate genes [46]. However, after correcting for multiple tests on each gene, 6% of these paralogs have an asymmetric rate of nonsynonymous substitution, and a joint analysis of 14% of these paralogs also supports significant asymmetry, an observation that is consistent with neofunctionalization. One explanation for a different number of nonsynonymous substitutions in each paralog is that each diploid ancestor of X. laevis had a substantially different effective population size and that this introduced unequal levels of polymorphism in alternative paralogs of the allopolyploid ancestor of X. laevis. But this scenario is not supported by the data: paralogs with significantly different rates of nonsynonymous substitution do not have significantly different rates of synonymous substitution (Table S3, Figure 5A and 5B).

Other studies have recovered conflicting results with respect to whether paralogs have a different [38,43,66–68] or have a generally similar [18,19] rate of nonsynonymous substitution. One way that asymmetry in nonsynonymous substitutions could be realized is via positive selection on one paralog. Accounts of positive selection have been found in many individual duplicated genes [65,69–72], but based on a branch-specific test over all sites, this study found only two out of 580 individual paralogs with a *ka/ks* ratio over one, and in both cases *(c-jun* and *deleted in colorectal cancer tumor suppressor),* the ratio was very close to one (Table S3). That this ratio is generally below neutral expectations suggests that neither copy is superfluous; selection is maintaining expression of both, either to preserve advantageous unique functions or to preserve redundant functions. Because the *ka/ks* ratio over all sites is a conservative estimate of the frequency of positive selection, we cannot rule out a role for site-specific positive selection in generating an asymmetric rate of nonsynonymous substitution in some paralogs. Comparison of the number of retained genes in species with different population sizes, for example, supports a role for positive selection in duplicate gene retention although it is not clear whether this is due to changes of amino acid sequences or regulation [73].

Rates of nonsynonymous substitution are also correlated with levels of expression [32–34], and one way that mutations could be sequestered to only one of the two paralogs is if regulation diverged prior to the accumulation of different numbers of substitutions. In *Saccharomyces cerevisiae,* for example, highly expressed paralogs evolve more slowly than paralogs with low expression levels [35]. Asymmetric rates could also be realized through enhancement or degradation of differently sized functional domains in each paralog. It will be interesting, therefore, to combine these results with information on expression to further evaluate the role of neofunctionalization versus other mechanisms in promoting the retained expression of these paralogs.

### Low Incidence of Complementary Replacement Substitutions

Only 3% of these paralogs were identified with complementary patterns of substitution, and this could be due to multiple factors. If retention is promoted by a small number of complementary mutations, mutations in the same (or nearby) positions, or splice variants, then the tests that we used would lack power. Additionally, amino acid substitutions in the diploid ancestors prior to allopolyploidization or near-neutral substitutions in either paralog after allopolyploidization could obscure an otherwise complementary pattern of substitution that occurred after allopolyploidization. That the estimated frequency of complementary substitutions is much lower in X. laevis than in paralogs shared by humans and mice [43] suggests that subfunction specialization or degeneration in the coding region is more prevalent in much-older expressed duplicates. Substantive changes in functional domains of each paralog may occur more commonly in older duplicates, for example after regulatory changes have occurred [74].

### Retention of Genes with Overlapping or Redundant Functions

That we did not detect a significant difference between the *ka/ks* ratio of singletons and paralogs of most genes suggests that changes in functional constraints after duplication by allopolyploidization are often small. The degree to which retained expression of most of these paralogs (78%), whose evolution was not significantly different from a singleton ortholog, is attributable to mechanisms that do not necessitate changed functional constraints depends on the level of Type II error of the tests that we deployed. Some paralogs have a lower *ka/ks* ratio than the diploid lineage and, under assumptions and caveats discussed earlier, functional constraints of these loci either did not change or even became more extreme after duplication.

Many scenarios exist in which these paralogs could be retained without substantial changes in the *ka/ks* ratio, and our results indicate that this class of mechanisms is pervasive in the first dozens of millions of years of duplicate gene evolution. Selection to maintain stoichiometry, selection for overexpression, and quantitative, temporal, or spatial subfunctionalization (Figure 1) preserve paralogs with identical function. Functional differences can be achieved by a small number of amino acid substitutions [75,76] and a small number of activity-reducing substitutions could sufficiently impair function of both paralogs to the extent that both are required [60].

### Multiple Mechanisms

We have used a simple paradigm to associate duplicate genes to nonoverlapping categories of retention mechanism, although in reality there is reason to believe that a combination of factors may operate on a single duplicate copy. A functional study of *Sa. cerevisiae* indicates that multiple mechanisms promote the retention of duplicate genes and that these mechanisms sometimes collaborate to promote retention of the same paralogs [77]. Evidence from X. laevis also supports this notion. Duplicated copies of the estrogen receptor α, ERα1 (AY310906) and ERα2 (AY310905), for example, exhibit signs of a combination of types of subfunctionalization in *X. laevis:* ERα2 is missing the N-terminal domain, and splice variants of each paralog are expressed in different tissues [78]. A combination of mechanisms is also suggested by *X. laevis embryonic fibroblast growth factor* (EFGF), which is a secreted protein with mesoderm-inducing activity: one X. laevis EFGF paralog (X62594) has five out of six amino acid mutations in a hydrophobic signaling domain, part of which gets cleaved after expression, whereas the other paralog (X62593) has seven out of eight substitutions and a four amino acid deletion in a different domain that elicits fibroblast growth factor activities (Figure 4C) [79]. These paralogs also have divergent timing and stoichiometry of expression [79]. Likewise, both nonsynonymous substitutions of one paralog (U05003) of the *FTZ-F1–related nuclear receptor* gene are in the “E domain III”, which is involved in dimerization and transcriptional activation or suppression, whereas the other paralog (U05001) has eight substitutions including two in an otherwise highly conserved zinc finger–containing C-domain that is responsible for DNA binding and one substitution in the FTZ-F1 box (Figure 4D) [80]. These paralogs are also differently expressed during embryogenesis [80].

### Conclusions

Thus, evolution of some paralogs (6%) is consistent with neofunctionalization in that they have different rates of nonsynonymous substitution with one of them evolving faster than a singleton; this is most obviously suggested by substitutions in paralogs of *liver-type arginase*. There remains a lack of consensus regarding the significance of neofunctionalization [18,19,38,43,66,67], and further characterization of the expression domains of these asymmetrically evolving paralogs is of interest. With the caveat that substantial functional transitions could be achieved by a small number of amino acid changes, complementary degeneration or enhancement of complementary protein functional domains appears rare in these relatively young paralogs (~30 million years old). Functional constraints on most of these paralogs are similar to homologous singletons. Synthesis of molecular evolution and expression of these paralogs indicates that multiple mechanisms operate sequentially or concurrently to promote their expression within the same genome, in genes of the same functional class, and over the same period of time following duplication.

## Materials and Methods

### Identification of paralogs.

We used multiple approaches to test whether X. laevis sequences were derived from genome duplication (tetraploidization) as opposed to another gene duplication event, and to test whether these sequences were paralogous rather than allelic. Outgroup sequence from another amphibian, a reptile, a mammal, or a fish were selected from Genbank in order to maximize the number of bases with unambiguous homology and phylogenetic proximity to clawed frogs. A rooted genealogy of the X. laevis paralogs, the S. tropicalis ortholog(s), and the outgroup was estimated using maximum likelihood with PAUP* [81] and a model of substitution selected with Modeltest version 3.06 [82]. We included X. laevis paralogs that formed a clade with respect to the S. tropicalis ortholog, as expected because tetraploidization of X. laevis occurred after divergence of *Xenopus* and *Silurana* [26]. We excluded genes that were duplicated in *Silurana* after the divergence of *Xenopus*. To explore the possibility that the sequences were actually allelic variants of one gene rather than alleles of separate paralogous genes, we compared the patristic distance between X. laevis paralogs to the average patristic distance between each paralog and the S. tropicalis ortholog. We applied a rule of thumb based on our estimates of the divergence times, that X. laevis paralogs should be at least one third as divergent from each other as they were from the S. tropicalis sequence. One possibility that we could not rule out is that duplication of one of the paralogs occurred in X. laevis after tetraploidization, which would result in more than two post-tetraploidization paralogs in X. laevis. However, we expect this possibility to comprise a small portion of the genes that we analyzed, and to not substantially compromise conclusions drawn regarding the impact of gene duplication in X. laevis relative to a singleton ortholog in S. tropicalis. We included all genes analyzed by Hughes and Hughes [16] except *calmodulin* because our analyses suggested that these sequences are not derived from the tetraploidization of X. laevis.

We identified some expressed putative paralogs in which phylogenetic analysis did not recover the expected relationship between the *Xenopus* paralogs and a closely related S. tropicalis ortholog, but instead provided weak support for an alternative relationship (Table S1), even though these genes had only one closely related ortholog in the S. tropicalis genome, and the ratio of patristic distances was within our expectations. We used parametric bootstrapping [83,84] to test the null hypothesis that each of these genealogies is consistent with the expected topology depicted in Figures 2 and 3, and included those duplicates that did not reject this null hypothesis. For subsequent analyses without an outgroup, we sometimes included more data because homology within clawed frogs was unambiguous for all nucleotides.

### Models for the retained expression of duplicate genes.

We compared alternative models with different branch-specific *ka/ks* ratios or rates of nonsynonymous substitution [85] using the codeml program of PAML, version 3.14 [86]. One ratio or rate was estimated over all sites, and the transition/transversion ratio was estimated from the data. Equilibrium amino acid positions of the codon substitution model were calculated from the average nucleotide frequencies at each codon position. Significance of improvement in likelihood of the more parameterized model was assessed with a χ^2^ test with degrees of freedom equal to the difference in the number of free parameters of each model. We performed five independent estimations of the maximum likelihood of each model for each gene.

The baseml program of PAML was used to perform marginal reconstruction of the sequences of the node ancestral to the X. laevis paralogs based on a model partitioned by each codon position with a different transition/transversion rate ratio, different base frequencies, and branch lengths proportional for each partition. The ancestral reconstruction and the extant sequences were used to estimate the number and positions of synonymous substitutions with DNAsp, version 4.0 [87].

### Codon bias.

If codon bias is positively correlated with expression levels, the rate of synonymous substitution would be underestimated to a greater degree in duplicated genes, and this could inflate estimates of the post-duplication *ka/ks* ratio [88,89]. To explore this possibility, we compared the codon bias of each X. laevis paralog to the codon bias of the S. tropicalis sequence and a maximum likelihood reconstruction of the sequence of the diploid ancestor of X. laevis (Node 1 on Figure 2). Codon bias of each pair of sequences was quantified with the scaled χ^2^ statistic [88] as calculated by DNAsp. Significance of the partial correlation coefficients between this estimate of codon bias and number of extra gene copies (zero or one) was assessed while holding constant the impact of the number of synonymous substitutions on the branches connecting each pair of sequences [44,90].

### Equal means Skellam distribution.

We used an approach described by Lynch and Katju [44] to evaluate the null hypothesis of equal evolutionary rates. This test is based on a special instance of the Skellam distribution that describes the probability distribution of differences between two samples drawn from the same Poisson distribution [91]. For each pair of duplicates, the number of sites that experienced a nonsynonymous substitution or a synonymous substitution since divergence was estimated by comparing each sequence to the reconstructed ancestral sequence. This is a conservative estimate of the magnitude of the difference in evolutionary rates, because multiple substitutions in the same site are not counted.

In order to improve the statistical power for this test [46], we concatenated data from multiple loci into two “superparalogs.” A randomly chosen paralog from each locus was concatenated into one of the superparalogs, and the other paralog from each locus was concatenated into a second superparalog. The difference in the number of mutations in each superparalog (d_SP_) was then calculated, and superparalog construction was pseudoreplicated for 10,000 iterations to generate a probability distribution of d_SP_. Under the null hypothesis of equal rates of nonsynonymous substitution, this probability distribution approximates an equal mean Skellam distribution with the expected number of substitutions equal to the sum of the mean number of substitutions in each superparalog (λ_SP_). This is true because the sum of multiple Poisson distributions is a Poisson distribution with mean equal to the sum of the constituent distributions.

To evaluate significance, we compared the fit of the observed and simulated probability distributions of d_SP_ to the expected equal mean Skellam distribution. For each simulated locus, the number of mutations on each paralog was drawn from a Poisson distribution. The mean of this Poisson distribution was drawn from another Poisson distribution with a mean equal to the average number of substitutions at the locus being simulated. This approach accommodates uncertainty in the expected number of substitutions at each locus in the test, as well as stochastic sampling of the number of mutation from the distribution defined by this mean. Superparalogs were constructed out of the simulated paralogs, and a probability distribution of d_SP_ was obtained in the same way as for the observed data. Fit of the observed and 1,000 simulated probability distributions relative to the expected equal mean Skellam distribution were compared with the χ^2^ statistic; the median χ^2^ value from nine iterations of the observed data was used as the test statistic.

Because variation in the nonsynonymous substitution rate could stem from different evolutionary rates in different genomic regions rather than different functional constraints at the amino acid level, we excluded from the equal means Skellam test loci in which the number of synonymous substitutions in each paralog did not meet the Poisson expectation that the mean number of substitutions in each paralog equal the variance. This deviation could stem from variation in the genome-wide rate of evolution, sequencing errors, or other unknown factors, and this would confound efforts to test whether nonsynonymous substitutions have different evolutionary rates due to differential selection on nonsynonymous sites of each paralog. Substitutions in arginase, for example, are suggestive of different evolutionary rates that affect both classes of substitutions (Table S1), because one paralog has many nonsynonymous and synonymous substitutions whereas the other is identical to the reconstructed ancestral sequence (i.e., Node 1 in Figure 2). Significance of the departure of the variance in the number of synonymous substitutions from the Poisson expectation that it equal the mean was tested with a χ^2^ test with Yates correction for small sample size, a d value for infinite degrees of freedom and a liberal rejection criterion (α = 0.20). Using this criterion, we eliminated 32 of the 290 genes from this analysis (Table S1).

### Complementary substitutions in each paralog.

We used two methods to test whether substitutions occurred in complementary locations in each paralog. The first method is the paralog heterogeneity test of [43], which was derived from a test for heterogeneous substitution in a singleton protein [92,93]. We applied this approach to the two paralogs in X. laevis by considering new mutations in each paralog as opposed to variable mutations between paralogous pairs of orthologs [43]. Significance of the absolute differential of the longest region of different substitution heterogeneity between paralogs (“R” from [43]) was assessed by comparison to a null distribution of absolute differentials. This distribution was generated from 1,000 simulated paralogs with the same number of substitutions as the observed paralogs and the locations drawn from a permutated set of all observed variable sites in either paralog or in the diploid lineage. This is a more conservative approach than generating a null distribution of R values from a random assignment of mutations [43], because it assumes that substitutions in a homologous singleton are also heterogeneous.

The second test we used is the runs test for dichotomous variables [44], which tests whether substitutions occur adjacently on the same paralog more frequently than expected by chance. Mutations on each paralog were ordered and converted to a string of binary variables to indicate whether they were on the α or β paralog. We assumed that mutations in the same position on both paralogs interrupt a run. Significance was estimated as the rank of the observed number of runs relative to the number of runs in 100,000 permutations. The paralog heterogeneity test and the runs test were performed only on paralogs that both had at least three mutations. Perl scripts that perform these tests are available by request.

### Estimation of the age of X. laevis paralogs.

The age of genome duplication in *Xenopus* (between Nodes 1 and 3 in Figure 2) was estimated from nongapped sequences from two genes using a relaxed molecular clock with r8s version 1.7 [94,95]. To minimize the impact of duplication on our estimates, we analyzed only synonymous substitutions at fixed amino acid positions and four-fold degenerate synonymous substitutions in pipoid frogs. We included 302 variable sites from *RAG1* and 162 from *chemokine receptor 4*. Calibration points were obtained from fossil evidence (23.8 million years as a minimum age of *Xenopus* based on the derived morphology of the fossil species X. arabiensis [96] and geological evidence (112 million years for the separation of *Pipa* and *Hymenochirus* due to the separation of Africa and South America [97,98]. The maximum age of the root of the topology was limited to the age of the earliest frog fossil, 195 million years [99]. For *cytochine receptor 4,* we assumed that an unidentified species (AY523691) is either X. borealis, *X. muelleri,* or *X. “new tetraploid”* [48], thereby providing an estimate for Node 3 in Figure 2B. If this unknown species is actually another tetraploid with a closer relationship to *X. laevis,* the estimated time of this node would be younger than the actual age of Node 3 in Figure 2B. Confidence intervals were obtained by bootstrapping the data as in Evans et al. [48]; an appropriate outgroup *(Scaphiopus* or *Spea)* was used to root Pipoids and then pruned from the topology. Average dates and confidence intervals weighted by the number of variable sites analyzed from each gene are reported.

## Supporting Information

Table S1 Gene Information Information on genes including base pairs (bp) analyzed, accession numbers of X. laevis paralogs and outgroup, number of polymorphic nonsynonymous and synonymous sites on each paralog and the diploid lineage, and the expected (mean) number of polymorphic sites (λML)
(105 KB PDF)

Table S2 *ka/ks* Ratios Comparison of *ka/ks* ratio (ω) before versus after gene duplication using a branch test and across diploid and tetraploid lineages (Model A versus B in Figure 3)
(91 KB XLS)

Table S3 Nonsynonymous Substitution Rates Results of test for different nonsynonymous substitution rates in each paralog (Model B versus C in Figure 3).
(69 KB PDF)

Table S4 Tests for Complementary Patterns of Substitution Tests for complementary patterns of substitution using the paralog heterogeneity test and runs test for dichotomous variables on nonsynonymous and synonymous substitutions.
(52 KB PDF)

### Accession Numbers

Most of the Genbank (http://www.ncbi.nlm.nih.gov/Genbank) accession numbers for the nucleotide sequences analyzed in this paper are listed in Table S1. The Genbank accession numbers for nucleotide sequences specifically mentioned in the text of this paper are as follows: *calmodulin* (K01944) and (K01945); *cytochine receptor 4* (AY364174), (AY523685), (AY523691), (AY523699), (AY523701), (BC044963), (CR942369), and (Y17895); *estrogen receptor* α1 (AY310906); *estrogen receptor* α2 (AY310905); *fibroblast growth factor receptor* (M55163) and (U24491); *embryonic fibroblast growth factor* (X62593) and (X62594); *FTZ-F1–related nuclear receptor* (U05001) and (U05003); *liver-type arginase* (BC043635) and (X69820); *RAG1* (AY874301), (AY874302), (AY874303), (AY874305), (AY874306), (AY874315), (AY874328), (AY874341), and (AY874357); and *transcription factor XCO2* (AF041138).

## Abbreviation

mya: million years ago
