# Supplementary material for: Multiple Mechanisms Promote the Retained Expression of Gene Duplicates in the Tetraploid Frog Xenopus laevis
Source: PLoS Genet. 2006 Apr 28;2(4):e56. doi: 10.1371/journal.pgen.0020056 (PMC1449897; doi:10.1371/journal.pgen.0020056)
Supplement: Table S1 — Information on genes including base pairs (bp) analyzed, accession numbers of X. laevis paralogs and outgroup, number of polymorphic nonsynonymous and synonymous sites on each paralog and the diploid lineage, and the expected (mean) number of polymorphic sites (λML) (105 KB PDF) [file pgen.0020056.st001.pdf]

Supplementary Information Table 1: Information on genes including base pairs (bp) analyzed, accession numbers of *X. laevis* paralogs and outgroup, number of polymorphic nonsynonymous and synonymous sites on each paralog and the diploid lineage, and the expected (mean) number of polymorphic sites (λML). Note that the number of polymorphic sites is less than or equal to the number of substitutions because multiple substitutions could occur at a site. Genes analyzed by Hughes and Hughes (1993) are indicated with an asterisk after the name. Genes of which only partial coding sequences were analyzed are indicated with an asterisk after the bp analyzed. Accession numbers with an asterisk are *Silurana tropicalis* sequences obtained from Genbank instead of the genomic scaffolds. Genes not included in the equal means Skellam test due to overdispersion of synonymous substitutions have an asterisk after the synonymous (λML). If the expected rooted topology between X. laevis paralog a (XLA), b (XLB), *S. tropicalis*, and the outgroup was not estimated with phylogenetic analysis, we report the P values of a parametric bootstrap test of this null hypothesis; nonsignificant values indicate that the expected topology cannot be rejected by the data. Significance after Bonferroni correction for Analyses 1, 2 and 3 is identified identified by a "+"; P values for these tests are in Supplementary Information Tables 2, 3, and 4.

			replacement substitutions				synonymous substitutions					Analysis number		
Xenopus Gene Name	bp	Xenopus & Outgroup Accession#	α	β	Dip	λ_{ML}	α	β	Dip	λ_{ML}	Bootstrap P value	1	2	3
Actin (skeletal, alpha 3)*	1131	X12525<br>X03470<br>J00068	0	0	2	0	23	15	24	19				
Activin Receptor-Like Kinase-2 (ALK-2)	1455	AB001073<br>AF012245<br>AJ318064	9	8	5	8.5	37	29	56	33				
Activin receptor II	1530	BC077946<br>M88594<br>NM_204317	9	15	5	12	45	31	64	38		+		
Adipophilin (fatvg)	1194	AF184090<br>BC082351<br>XM_424822	22	15	18	18.5	30	28	39	29	0.105			
AE (Amidating Enzyme)	2610	X62771<br>M19032<br>XM_424857	24	30	26	27	71	71	83	71				
Albumin (serum)*	1809	M18350<br>M21442<br>U40452	38	29	88	33.5	39	20	85	29.5*				
ALDH (Aldehyde dehydrogenase class1)	1503	AB016717<br>AB016718<br>X58869	14	12	12	13	17	24	41	20.5				
Alpha Globin	426	X14259<br>X14261<br>M63797	3	6	23	4.5	12	8	15	10				
Amelogenin	489	AF095569<br>AF095570<br>AF095568	11	18	15	14.5	10	15	16	12.5				
Xenopus Anterior Neural Folds, Homeobox gene	555	X60099<br>U28370<br>XM_541834	11	10	13	10.5	14	20	27	17				
Amyloid-Beta-like protein precursor	2244	AJ608932<br>AJ608933<br>BC000373	30	13	10	21.5	39	43	56	41		+	+	
Apoptosis Inhibitor 5	1485	BC043906<br>BC077529<br>BC007133	6	11	6	8.5	39	46	78	42.5				
AR (Androgen Receptor)	471*	U67129<br>X58955<br>AY324231	0	2	3	1	0	5	20	2.5*				
Liver L-arginase	945	BC043635<br>X69820<br>D38303	0	25	25	12.5	0	19	64	9.5*		+	+	
Arginase Type 2	1080	U08406<br>U08408<br>AY074489	3	7	1	5	25	27	31	26				
Arrestin	1128	BC076815<br>BC072973<br>Z11501	10	7	6	8.5	32	34	58	33				
Aspartyl tRNA synthetase	1590	BC042227<br>BC072839<br>NM_001006528	7	4	9	5.5	39	35	90	37				
Atonal Homolog 5	414	U93170<br>U93171<br>AJ630209	9	4	5	6.5	7	10	11	8.5				
ATP synthase subunit B	750	AF187862<br>BC082702<br>AY522571	9	7	9	8	13	29	33	21*				
Bambi (TGF-beta signalling)	780	AJ243576<br>BC079987<br>BX934061	4	13	13	8.5	15	27	28	21				
Barren (brrn1, 13S condensin XCAP-H subunit)	2064	BC056095<br>BC068643<br>BC024211	23	24	23	23.5	51	59	76	55				
Bestrophin-2 (VMD2L1)	1530	AY273825<br>AY273826<br>NM_017682	25	32	31	28.5	28	37	75	32.5		+		
Beta Globin	438	J00978<br>BC071139<br>DQ091201	10	13	29	11.5	9	13	14	11				
Complement factor B (Bf B) (MHC class III gene)	2235	D29796<br>D49373<br>XM_532086	75	73	70	74	59	48	77	53.5				

		AB037269												
Biglycan	1098	BC074403<br>BT007323<br>AF224746	4	7	17	5.5	26	20	46	23				
Bicaudal-C	1908*	BC084957<br>XM_421490<br>BC046852	16	10	10	13	53	35	80	44				
Bridging integrator 1 (Amphiphysin II)	1389	BC073530<br>NM_001006524<br>BC078006	22	13	16	17.5	36	34	60	35				
Bing4	1632*	BC077337<br>AY648746<br>D32066	14	26	31	20	28	24	58	26				
BMP (Bone Morphogenetic Protein) receptor	1431*	D32067<br>BC028383<br>BC044074	6	7	10	6.5	35	33	44	34				
Block of proliferation 1	960*	BC056015<br>NM_015201<br>BC072031	5	12	28	8.5	15	11	61	13				
Brachyury (T)	1296	BC072224<br>AB001939<br>AB113401	6	12	9	9	28	30	51	29				
Serine/Threonine protein kinase (c-RMIL)	2217*	AY728383<br>X67052<br>U35408	11	17	19	14	43	63	104	53				
Basic transcription element binding protein	864	U35409<br>Y14296<br>AB028243	1	3	15	2	4	11	24	7.5				
B-cell translocation gene 1, anti-proliferative	507	BC041244<br>NM_205350<br>BC046715	8	6	3	7	5	8	15	6.5		+		+
Calcium homeostasis endoplasmic reticulum protein	2772	BC080042<br>NM_138585<br>BC044970	38	16	13	27	66	60	112	63		+	+	
Calnexin	1800	BC041719<br>D78590<br>BC046257	28	18	37	23	29	40	72	34.5				
Calponin H3 (clpH3)	876	BC073467<br>CR391451<br>BC046699	5	6	8	5.5	12	29	39	20.5*				
Calreticulin	1236	BC044068<br>D78589<br>BC042287	10	11	20	10.5	41	28	44	34.5				
Carbonic anhydrase II	780	BC041213<br>BC004897<br>Y08817	17	7	18	12	13	14	33	13.5				
Casein kinase I alpha S (Csnk1a1)	1011	BC043956<br>U80822<br>BC077777	0	1	0	0.5	23	21	13	22				
CASK interacting protein 2	3609	BC092148<br>XM_420128<br>BC044689	60	47	68	53.5	87	113	155	100				
Procathepsin B	999	BC046667<br>XM_429301<br>BC077191	7	13	15	10	26	32	38	29				
Beta Catenin interacting protein 1 (catnbip1)	243	BC082479<br>BC014300<br>BC077504	0	1	0	0.5	6	2	3	4	0.06			
Cystathionine-beta-synthase	669	BC044091<br>NM_178224<br>U33217	4	3	5	3.5	19	22	38	20.5				
voltage-dependent Calcium channel beta subunit	1443	U33218<br>BC041811<br>M60680	6	15	8	10.5	23	28	44	25.5				
CDC2 (cell division cycle 2, kinase)	906	M60681<br>AF159158<br>AB080684	3	9	1	6	25	27	43	26		+		
Cathepsin E	1191	AB080685<br>AB093036<br>BC054271	10	14	15	12	28	33	40	30.5				
Carboxyl ester lipase	1419	BC054227<br>NM_009885<br>BC078053	13	9	16	11	47	38	82	42.5				
Carboxyl ester lipase (bile salt-stimulated lipase)	1641	BC085225<br>NM_001012997<br>U37538	20	22	33	21	42	56	97	49				
Centrin	516	BC054948<br>NM_019405<br>BC054217	2	1	4	1.5	11	8	12	9.5				
Cerebellin 2 precursor protein	645	BC074247<br>NM_172633<br>X59958	9	12	4	10.5	11	13	22	12		+		
Complement factor I (C3b/C4b inactivator)	1755	BC041753<br>Y18965<br>U60209	53	35	50	44	36	40	53	38				
Cystic fibrosis transmembrane conductance regulator	4407	X65256<br>AY026761<br>X82626	18	21	54	19.5	41	33	184	37		+		
Cortical granule lectin	939	BC085052<br>AY157364	15	22	26	18.5	26	40	44	33				

Choroideremia (Rab escort protein 1)	1884	BC061662<br>BC078011<br>NM_017067	22	45	41	33.5	39	41	42	40	0.12		
Carbohydrate sulfotransferase 11 (Chst11)	993	BC056117<br>BC089137<br>XM_414372	7	4	6	5.5	22	20	31	21			+
Cell death-inducing DFFA-like effector c (CIDE-3alpha)	708	BC077471<br>BC082372<br>AY364640	10	9	14	9.5	15	22	33	18.5			
C-Jun proto-oncogene (AP-1, Activator Protein)	936	AJ243955<br>AJ243954<br>X15547	12	13	14	12.5	5	21	37	13*		+	
Dipeptidase 2 (metallopeptidase M20 family)	1422	BC075171<br>BC056069<br>NM_001006385	17	10	12	13.5	31	24	52	27.5			
alpha-1 Collagen type II	4458	M63595<br>M63596<br>AB022046	22	39	30	30.5	48	67	86	57.5			
Connexin 31 (Gap junction beta-3 protein)	801	AY057997<br>BC072821<br>NM_001009780	11	7	4	9	20	14	30	17		+	
Contactin/F3/F11 (Contactin A)	3015	D86505<br>AB015205<br>NM_001004381	39	43	30	41	56	62	95	59		+	
Coronin	1440	AB025245<br>AB025246<br>AK223073	16	15	15	15.5	33	31	42	32			
Cortactin	1590	AB027611<br>BC073454<br>BC011434	18	15	26	16.5	46	39	69	42.5			
Cytoplasmic polyadenylation element binding protein	1704	U14169<br>BC077702<br>AF329403	13	7	6	10	38	36	52	37			
CRY2 (cryptochrome 2)	1503*	AY049034<br>AY049035<br>AY256684	22	18	22	20	46	59	89	52.5			
Crystallin, beta A1	633	BC053794<br>BC082450<br>X87759	11	6	5	8.5	8	6	11	7			
Cathepsin S (CTSS)	999	BC056059<br>BC077285<br>M90696	16	17	25	16.5	14	23	37	18.5			
Cullin3 (Cul3)	2304	BC077239<br>BC073186<br>AF129738	1	2	0	1.5	46	56	65	51			
CyclinE	1224	L23857<br>L43513<br>U28981	22	14	14	18	31	28	27	29.5	0.22		
Brain Dopamine receptor D2	1035*	X59500<br>X72902<br>X17458	9	13	6	11	24	19	18	21.5			
Dapper 1, antagonist of beta-catenin (Frodo)	2454	BC077380<br>AF393622<br>NM_016651	33	51	23	42	52	89	74	70.5*			
Death-associated protein kinase 1	4281	BC077360<br>BC070744<br>X76104	19	33	16	26	77	87	133	82		+	
Drebrin-like	1125	BC044296<br>BC074277<br>NM_013810	21	22	17	21.5	29	15	40	22			
Debranching enzyme homolog 1	1596	BC046698<br>BC073223<br>NM_031403	26	14	16	20	44	34	59	39			
Deleted in colorectal cancer tumor suppressor	486*	U10986<br>U10954<br>X76132	5	14	10	9.5	6	7	26	6.5		+	
Desmin	1374	X16842<br>BC077922<br>AB011672	13	18	10	15.5	33	26	66	29.5		+	
Hand2	396*	AF286645<br>AF286646<br>NM_133803	1	5	0	3	6	8	10	7		+	
Cytoplasmic dynein light-intermediate chain 1 (DLIC1)	1524	AF317841<br>BC045030<br>AK222653	11	12	8	11.5	29	29	36	29			
Dipeptidylpeptidase 3	2187	BC060495<br>BC077244<br>NM_133803	36	33	36	34.5	68	37	80	52.5*			
Dullard	732	AB084264<br>BC082639<br>XM_536616	0	2	2	1	15	7	48	11			
Dystroglycan (DAG1)	2655	BC046260<br>BC073500<br>XM_343483	24	16	35	20	60	55	68	57.5			
Dystrophin	1761*	X99700<br>BC082429<br>NM_004007	4	0	5	2	11	0	57	5.5*			
Helix-loop-helix transcription factor XE1	441	U25959<br>U25960<br>M83233	4	7	3	5.5	7	9	15	8			

E2 (transcription factor E2)	1962	BC044064<br>X66959<br>NM_003200	25	8	15	16.5	42	27	54	34.5			+	
met-mesencephalon-olfactory transcription factor 1 (Ebf2)	1713	AF040993<br>AF041138<br>U71189	11	19	2	9.5	25	37	58	31		+		
CCAAT/enhancer binding protein (C/EBP), alpha	891	BC041714<br>BC072168<br>U08604	7	19	21	15	13	25	35	19				
Endothelin receptor type A	1242	BC044316<br>U06633<br>NM_204119	14	9	12	11.5	24	44	46	34*				
EF (Elongation Factor-1 alpha, 42Sp48)	1362*	X52976<br>X52977<br>AB124568	2	7	2	4.5	32	39	52	35.5				
Aurora kinase A (EG2)	1218	Z17206<br>Z17207<br>BC005425	8	16	29	12	37	43	69	40				
Engrailed 2 (EN2)	789	X62973<br>X62974<br>NM_010134	3	8	18	5.5	14	15	23	14.5				
Enkephalin A (proenkephalin A)*	672	X00852<br>X00853<br>AF195886	8	4	4	6	8	10	19	9				
ENO (2-phosphoglycerate dehydratase, enolase)	1302	BC054169<br>Y00718<br>D37900	0	6	24	3	37	26	55	31.5				
Era (Estrogen Receptor alpha)	1281	AY310906<br>AY310905<br>AB055221	3	8	8	5.5	17	32	37	24.5*				
Enhancer of split groucho	2268	BC077170<br>AF289027<br>AF186092	0	5	9	2.5	34	33	92	33.5				
Enhancer of zeste	2244	AF351126<br>BC097526<br>XM_418879	8	19	2	13.5	44	53	46	48.5		+		
Focal adhesion kinase	3147	L33920<br>BC071046<br>M86656	10	20	20	15	45	76	128	60.5*				
Transcription factor (XLFB1)	321*	X64759<br>X64760<br>NM_000545	4	5	1	4.5	4	8	4	6				+
XFD-4	1377	AJ249225<br>AJ249224<br>Y08223	13	16	8	14.5	14	29	41	21.5*		+		
Flap endonuclease-1	1146	AF036327<br>U68141<br>X76771	9	9	14	9	28	46	52	37*				
FetuinB	1326	BC043891<br>BC078490<br>XM_422765	36	43	57	39.5	28	41	35	34.5				
Ftz-F1-related orphan receptor (xFF1r)	1392	U05001<br>U05003<br>AB035498	8	2	2	5	23	23	36	23				
FGF (embryonic fibroblast growth factor 4)	561	X62593<br>X62594<br>U76998	8	6	10	7	14	4	13	9				+
Fibroblast growth factor receptor	2436	M55163<br>U24491<br>X59380	15	27	13	21	49	54	93	51.5		+	+	+
Fibrinogen alpha	2244	BC041754<br>BC073502<br>U44830	26	52	46	39	43	38	60	40.5			+	
Flotillin	1254	AF545659<br>AF545661<br>NM_005803	16	17	13	16.5	25	29	45	27	0.125			
fms-related tyrosine kinase 1	2043	BC044264<br>BC056023<br>XM_534520	50	48	53	49	36	55	73	45.5				
Fms-interacting protein (NF2/meningioma region)	2034	BC045075<br>BC076720<br>NM_001006195	25	19	14	22	42	51	91	46.5		+		
alpha-fodrin (Xen alpha 1) spectrin, non-erythrocytic 1	705*	X06041<br>X06042<br>BC034956	6	8	7	7	20	11	31	15.5				
c-fos proto-oncogene	261	AJ224511<br>BC079689<br>X06769	2	5	4	3.5	3	5	7	4				
Succinate dehydrogenase complex, Flavoprotein	1995	BC047261<br>BC060446<br>XM_419054	18	13	18	15.5	33	44	60	38.5				
Frequenin	570	U27274<br>BC072084<br>X84048	1	1	0	1	16	14	23	15				
Fascin	1446	BC077847<br>BC097600<br>AY044229	18	8	19	13	50	31	90	40.5				
Furin*	1779	M80471<br>AY901983<br>X54056	7	14	17	10.5	35	37	65	36				

Fused toes homolog	876	BC077318<br>BC084320<br>NM_001005838	4	8	4	6	16	21	20	18.5	0.5			
FYN (proto-oncogene c-fyn)	1608	BC043749<br>M27502<br>NM_002037	2	2	2	2	23	27	53	25				
Galectin	858	AB060970<br>BC081109<br>X79303	14	16	14	15	18	19	34	18.5				+
alpha subunit of Gq Gtp-binding protein (G protein)	1077	L05540<br>BC081126<br>U43083	3	1	5	2	14	13	22	13.5				
GATA-binding protein transcription factor GATA-1	1077	M76566<br>M76563<br>NM_002049	20	23	20	21.5	22	35	47	28.5				
Transcription factor xGata5	1155	L13701<br>L13702<br>NM_205421	11	6	15	8.5	26	21	68	23.5				
Growth hormone A	417*	AF193797<br>X14601<br>M33697	2	8	4	5	11	11	20	11				
Guanylate kinase 1	594	BC078071<br>BC077482<br>XM_425960	10	5	14	7.5	7	12	34	9.5				
Glycogenin 1 (mitotic phosphoprotein 45)	924	BC077236<br>AF419148<br>NM_001006558	12	16	10	14	23	36	31	29.5				
Holocytochrome c synthase (heme-lyase) (hccs-prov)	897	BC045005<br>BC078076<br>AJ851811	11	27	19	19	18	15	26	16.5			+	
cephalic Hedgehog, sonic hedgehog protein 4	1188	U26349<br>U26350<br>NM_009170	13	19	17	16	23	29	36	26				
Transcription factor XHEN1	384	U94992<br>BC084434<br>XM_424510	3	3	6	3	10	7	24	8.5				
Hypoxia-inducible factor 1 alpha	1461*	AY189821<br>BC043769<br>NM_204297	17	10	7	13.5	32	23	43	27.5		+		
SafA - scaffold attachment factor A	2313	AF068847<br>BC072816<br>XM_419539	41	26	34	33.5	71	57	104	64				
Homeobox 2/2.3*	474*	X06592<br>M23916<br>M16937	5	3	8	4	9	3	19	6				
Insulin*	318	M24442<br>M24443<br>V00565	3	5	1	4	5	6	9	5.5				
Integrin beta-1 subunit*	2394	M20140<br>M20180<br>X07979	9	10	17	9.5	58	74	92	66				
Inversin	2982	AF321228<br>AF321229<br>NM_204551	73	58	91	65.5	80	74	139	77				
Ubiquitin carboxyl-terminal hydrolase 5 (Isopeptidase T)	2562	BC079778<br>AY376839<br>U47927	19	16	15	17.5	44	57	84	50.5				
Kf-1 protein (Adgr34)	2001	AB083246<br>AB083247<br>AF306394	24	26	23	25	41	53	79	47				
Kit receptor tyrosine kinase (c-kit)	2739*	BC061947<br>Z48770<br>X06182	55	52	56	53.5	76	42	108	59*				
Kinesin-like protein 2	2712	X94082<br>BC071083<br>AB035898	25	23	33	24	45	54	76	49.5				
L1 (ribosomal protein L1)	1173	X05216<br>X05217<br>NM_001007479	7	1	5	4	24	42	36	33*				
L14 (ribosomal protein L14)	564	X06222<br>X06223<br>BC021743	4	5	6	4.5	9	12	12	10.5				
Lamin B	1749	AF077838<br>X06344<br>M34458	23	25	28	24	42	53	66	47.5				
Lamina associated polypeptide 2	1548	Y17861<br>AF048817<br>BC053675	31	22	45	26.5	33	20	43	26.5				
Clathrin, light polypeptide (Lcb)	615	BC077312<br>BC060412<br>AJ720113	9	6	3	7.5	17	15	19	16	0.32			
Lactate dehydrogenase	1002	U07179<br>U07176<br>XM_534868	11	9	24	10	32	18	41	25				
LEF-1 (lymphoid enhancer factor)	1116	AF287147<br>AF287148<br>AF288571	7	8	2	7.5	15	14	27	14.5		+		
TGF-beta family member Lefty-A	1032*	AF283562<br>AF283563<br>NM_130960	11	6	29	8.5	25	18	63	21.5				

LIM domain binding protein	1125	U74360<br>BC044043<br>BC013624<br>BC074438	0	1	0	0.5	16	18	14	17	0.115			
Lipocalin (Ptgds)	447*	BC074439<br>L06806<br>AJ249843	7	3	13	5	7	6	21	6.5				
Lpa1R (lysophosphatidic acid receptor)	1098	AJ249844<br>U70622<br>BC053822	4	2	0	3	16	15	26	15.5		+		
LR (Leptin Receptor)	393	BC078594<br>BC056250<br>AF276084	3	2	1	2.5	9	6	14	7.5				
Lipoprotein (LDL) receptor-related protein 6	636*	AF508961<br>AF074265<br>X68817	2	2	5	2	19	14	44	16.5				
Autoantigen La (La protein)	1275	X68818<br>BC081780<br>BC044036	21	23	34	22	39	30	48	34.5				
Microfibrillar-associated protein 1	1320	BC090193<br>BC050742<br>AF187864	9	9	7	9	28	36	57	32				
Myristoylated alanine-rich C kinase substrate	561	BC084888<br>NM_205480<br>L09738	20	9	14	14.5	16	13	23	14.5				
XMax2 and XMax4	381	L09739<br>NM_001009866<br>AY046531	1	0	1	0.5	6	3	4	4.5				
Myogenin	705	AY046532<br>XM_547345<br>BC042928	4	6	1	5	12	15	16	13.5				
Myozenin1	933	BC054224<br>XM_573691<br>M25696	22	15	12	18.5	20	23	21	21.5				
N-CAM (neural cell adhesion molecule)*	3252	M76710<br>D85084<br>U85969	51	30	46	40.5	59	51	75	55			+	
NF-M1 (middle molecular neurofilament)	2619	U85970<br>NM_005382<br>U67778	31	35	38	33	52	42	89	47				
neurogenin-related 1 (X-NGNR-1)	594	U67779<br>NM_204796<br>BC077476	11	14	21	12.5	15	20	31	17.5				
Internexin neuronal intermediate filament protein	1380	M86653<br>NM_199534<br>BC047968	21	13	18	17	33	23	58	28		+		+
NK3 transcription factor related, koza	633	AF127225<br>BC074863<br>BC057729	16	16	22	16	11	14	25	12.5				
Nonmuscle myosin II heavy chain A	2655*	AF055895<br>NM_022410<br>L09740	14	11	17	12.5	48	54	80	51				
Nonmuscle myosin heavy chain B	1062*	L11231<br>NM_175260<br>X05496	10	1	9	5.5	22	29	56	25.5			+	
Nucleolar-localized protein NO38	882	X56039<br>NM_205267<br>BC041205	9	22	18	15.5	22	22	29	22				
Nucleobindin 1	630*	BC068842<br>AF450266<br>BC068668	2	4	1	3	9	20	11	14.5*	0.55			
Nucleoplasmin	585	X04766<br>BC068078<br>AJ617672	5	7	12	6	14	17	19	15.5				
Nucleoporin (Nup88)	2178	AJ617673<br>Y08612<br>AY188504	19	34	36	26.5	54	58	84	56				
OLPA (Dorphin)	696*	AY188503<br>AY188505<br>AJ010978	16	19	17	17.5	16	20	47	18		+		
Olfactory marker protein (XOMP)	474	AJ010979<br>NM_011010	8	6	16	7	13	7	29	10				
OncogenesC-ets-1 (c-ets-1 proto-oncogene)*	1311	X52692<br>BC075161 (used instead of X52691)<br>J04101<br>M81683	5	4	5	4.5	29	37	29	33				
OncogenesC-ets-2 (c-ets-2 proto-oncogene)*	1392*	X52635<br>J04102<br>BC041189	15	13	10	14	35	48	67	41.5				
OncogenesC-myc (myelocytomatosis)*	1263	X56870<br>V00568<br>BC044069	11	13	10	12	30	20	54	25				
Dynactin 2 (p50)	1206	BC081081<br>AK222693<br>AJ277159	9	7	10	8	42	27	37	34.5				
PACSIN2	1026*	BC085213<br>CR456536	19	11	15	15	26	22	57	24		+		

Convertase PC2	1842*	X66493<br>BC074270<br>AB105176<br>AF239760	8	12	3	10	30	54	70	42*		+		
Prolyl isomerase (Pin1)	477	BC077181<br>BX950453<br>AB109739	3	3	5	3	9	15	18	12				
PKC (protein kinase C,delta)	2049	AB109740<br>BC043350<br>M95593	17	20	19	18.5	53	38	84	45.5				
Plakoglobin	2205	BC068787<br>Z68228<br>BC077880	11	12	15	11.5	57	59	111	58				
Peripheral myelin protein 22	474	BC041727<br>NM_008885<br>X03843	5	2	9	3.5	13	11	23	12				
POMC (pro-opiomelanocortin)*	777	X03844<br>AF115251<br>X59056	10	11	8	10.5	12	32	42	22*				
POU domain Gene 1	1068	BC093556<br>NM_131161<br>X64835	11	2	2	6.5	17	13	30	15		+	+	
POU3	840*	X96423<br>XM_539052<br>BC043624	7	1	7	4	10	9	19	9.5				
Phosphorylase phosphatase (Ppp2B)	1767	BC073612<br>CR860766<br>BC077952	7	1	1	4	26	49	64	37.5*				
Protein phosphatase 4, regulatory subunit 2 (Ppp4r2)	1191	BC088694<br>NM_174907<br>AF387815	26	31	22	28.5	21	43	37	32*				
LIM protein Prickle	2484	AY055473<br>XM_416036<br>AF193800	25	17	21	21	42	51	76	46.5				
Prolactin Receptor	1821	AF193801<br>AB158367<br>BC054174	38	42	37	40	41	54	66	47.5				
Prothymosin	324	BC045213<br>AJ312835<br>BC047245	7	6	1	6.5	6	8	5	7				
Phosphorylase, glycogen; brain	2529*	BC072163<br>BC030795<br>BC074233	18	15	14	16.5	45	74	96	59.5*				
RAB18 (member RAS oncogene family)	615	BC056054<br>AY357728<br>AF174644	2	6	2	4	13	15	18	14				
Rac GTPase	576	BC092101<br>AY279384<br>D38488	1	2	0	1.5	10	13	18	11.5				
Rad51	1008	D38489<br>AB020740<br>AY874341	4	2	2	3	29	32	49	30.5				
Rag-1	1134*	AY874315<br>AY874303<br>AJ304845	8	10	13	9	28	25	55	26.5				
Ral interacting protein (rlip) - RalA (RalB-binding protein)	1833*	AJ252165<br>AB209924<br>Y16259	5	16	11	10.5	30	39	43	34.5	0.53		+	
RalB	570	BC072046<br>X15015<br>X87365	2	0	8	1	18	12	61	15				
Retinoic acid receptor alpha	1185	L11445<br>X73972<br>L79913	5	18	8	11.5	37	16	71	26.5*		+	+	
RDS35 (retinal degradation slow/peripherin)	1035	L79914<br>AF031238<br>L79915	11	23	9	17	19	33	28	26	0.16			
RDS38/peripherin	972	BC054965<br>J02884<br>AB021737	19	4	9	11.5	20	18	44	19		+	+	
Requiem	1155	BC082478<br>XM_341998<br>BC054145	10	11	3	10.5	16	33	32	24.5*				
Rhodopsin	1062	L04692<br>U59922<br>AJ133499	1	5	13	3	6	5	25	5.5				
Ringo (p33 ringo, ls26)	888	AJ133500<br>XM_128768<br>BC077472	20	11	15	15.5	33	13	31	23*				
RIO kinase 2	1626	BC084165<br>NM_001006581<br>BC080086	28	28	23	28	40	36	52	38				+
Rwdd1 (RWD domain containing 1)	717	BC073326<br>AJ720663<br>AF001048	15	9	11	12	16	14	30	15				
Retinal homeobox A	966	AF001049<br>BC058757<br>BC073179	5	9	10	7	22	23	35	22.5				
Rxrb (retinoid X receptor beta)	1335	BC072132<br>BC001167	4	7	6	5.5	23	19	29	21	0.13			

Sister chromatid cohesion establishment factor (SCC2)	759	AY661732<br>AY661733<br>BC063859<br>BC071080	7	6	8	6.5	15	9	17	12			
Syndecan 2 (heparan sulfate proteoglycan 1)	570	BC041490<br>NM_001001462<br>BC043626	5	4	5	4.5	9	6	26	7.5			
Sek-1 receptor tyrosine kinase	2958	X91191<br>X65138<br>BC076643	7	8	2	7.5	35	50	109	42.5		+	
Selenoprotein I	1101*	BC071114<br>BC021229<br>BC043894	12	18	29	15	16	31	100	23.5*		+	
Selenoprotein T	414	BC044996<br>AY358096<br>BC077941	3	4	0	3.5	6	11	11	8.5		+	
Septin 11	981*	BC073250<br>XM_420341<br>AF212298	6	6	3	6	22	24	19	23			
Septin A (XlSeptA)	1056	BC082859<br>NM_010891<br>BC073077	3	3	0	3	21	16	31	18.5		+	
serum/glucocorticoid regulated kinase	1302	BC074305<br>NM_204476<br>AF279245	1	11	6	6	29	24	38	26.5			+
Shab12	540*	U20342<br>AF450111<br>BC046706	3	3	1	3	10	14	13	12			
Siah-interacting protein	678	BC074445<br>NM_009786<br>X68683	13	8	8	10.5	10	9	25	9.5		+	
Sloan-Kettering viral oncogene homolog	2145	U89999<br>M28517<br>U75681	15	13	13	14	38	26	75	32		+	
Histone stem-loop binding protein (SLBP)	762	BC073461<br>BT007433<br>AF390895	14	8	23	11	18	17	25	17.5			
Suc1-associated neurotrophic factor target	1527	BC046943<br>AF036717<br>D83650	16	19	11	17.5	33	26	31	29.5			
Sox11 (XLS13)	1041	D87209<br>NM_205187<br>AJ001730	9	13	21	11	23	11	57	17			
Sox17a (HMG box transcription factor Sox17-alpha)	1122	AB052691<br>NM_022454<br>AB052692	12	25	13	18.5	25	27	26	26	0.56		
Sox18 (Transcription factor SOX-18)	897*	AB052693<br>NM_204309<br>AF394958	9	12	20	10.5	15	23	54	19			
SP22	489*	DQ406635<br>AB073864<br>X62483	2	3	11	2.5	15	11	22	13			
Sparc	897*	BC045013<br>AB116365<br>BC077749	14	6	5	10	18	18	34	18		+	
Spats2 (spermatogenesis associated, serine-rich 2)	1641	BC057748<br>NM_139140<br>BC078118	32	13	24	22.5	29	42	53	35.5			
Spermatid perinuclear RNA binding protein	1338*	BC089298<br>BC017732<br>AF331824	29	16	14	22.5	33	26	40	29.5			
Sprouty-2	921	AF331825<br>AF176904<br>BC056037	13	8	7	10.5	17	14	20	15.5			
Sulfide quinone reductase-like	1329	BC054152<br>BC028247<br>M24704	14	15	8	14.5	38	24	45	31			
Src (pp60c-src protein)	1596	M23422<br>V00402<br>BC076749	6	5	3	5.5	33	26	69	29.5		+	
Stanniocalcin 1	741	BC084424<br>XM_425760<br>BC060382	13	7	10	10	21	15	36	18			
Staufen 1	1281*	AY705672<br>NM_001012831<br>BC046709	15	11	12	13	32	27	42	29.5			
Stress-induced-phosphoprotein 1	1620	BC078016<br>U27830<br>BC042356	18	21	12	19.5	32	47	53	39.5		+	
Stomatin	420*	BC054307<br>NM_004099<br>AF387816	4	2	3	3	11	17	16	14			
Strabismus	1563	AY069979<br>NM_020335<br>X81986	6	0	1	3	43	33	60	38			+
SUG1	1200	BC045221<br>XM_425834<br>AF467942	2	1	1	1.5	39	29	47	34			
translation initiation factor SUI1	339	BC054139<br>XM_534229	0	0	0	0	5	4	7	4.5			

Sumo	303	Z97073<br>BC090210<br>XM_516035<br>AB197247	3	0	0	1.5	3	4	4	3.5		
Survivin (Xsvv1)	480	AF442492<br>AB182320<br>AF035016	4	3	6	3.5	12	8	14	10		
Synaptobrevin	339	AF035017<br>NM_009497<br>AF035014	3	0	2	1.5	4	6	12	5		
Synaptophysin	882	AF035015<br>BC064550<br>AF059570	11	9	4	10	16	21	20	18.5		
Xwnt8 inhibitor sizzled (szl) (putative wnt inhibitor frzb3)	840	AF136184<br>AF308868<br>AB022691	9	4	11	6.5	19	10	24	14.5		
TAF-Ibeta	828	AB022692<br>AJ851581<br>AF133036	2	2	2	2	15	9	14	12		
T-box transcription factor Tbx5	360*	AB032944<br>U64433<br>AF499688	1	1	0	1	5	9	16	7		
TCRzeta subunit	477	BC098961<br>NM_206879<br>BC047131	13	7	16	10	18	11	21	14.5		
Bax Inhibitor-1, testis enhanced gene transcript	711	BC079707<br>BC005588<br>BC073440	10	8	3	9	10	14	27	12		+
TRK-fused protein TFG	1188	BC044079<br>U94662<br>M35343	8	10	8	9	26	30	25	28	0.18	
Thyroid Hormone Receptor alpha*	1254	M35344<br>L06064<br>M35359	2	6	4	4	11	16	33	13.5		
Thyroid Hormone Receptor beta*	1107	M35361<br>L27344<br>Y14446	3	5	1	4	18	16	18	17		
Mesoderm Posterior (Mesp)	924	Y14447<br>Y17043<br>AJ416632	20	18	14	19	26	23	17	24.5		
cytotoxic granule-associated RNA binding protein (TIA1)	1164	BC080105<br>NM_022173<br>AJ416631	8	3	2	5.5	32	25	36	28.5		
TIAR	1167	BC045086<br>NM_003252<br>M64659	6	10	3	8	31	19	38	25		
Tyrosine kinase	468*	M64660<br>M69243<br>M64661	4	6	0	5	15	7	15	11	0.31	+
IGF (Insulin-like Growth Factor) Receptor	462	AF055980<br>M69244<br>AB056893	1	3	2	2	6	6	17	6		
Transducer of erbB	894	BC078542<br>CR456594<br>BC054950	1	7	6	4	10	20	26	15		
Transferrin	2100	BC056840<br>U05246<br>M34699	42	28	63	35	52	37	86	44.5		
Thyrotropin-releasing Hormone	672	X64056<br>BC069375<br>AF305620	18	18	29	18	9	11	31	10		
Thyrotropin-releasing Hormone Receptor 1	1188	AJ420782<br>NM_003301<br>BC044959	7	3	9	5	17	18	68	17.5		
Neurotrophin receptor B xTrkB-alpha	1416	BC087462<br>X77251<br>AB003078	21	13	21	17	23	29	39	26		
fast skeletal Troponin C	483	AB003079<br>AB110088<br>AF441126	1	0	1	0.5	11	4	7	7.5		
unitary non-NMDA glutamate receptor subunit U1	1437	X93491<br>X17314<br>BC077923	14	11	14	12.5	35	30	59	32.5		
Ubiquiton-conjugating enzyme e2e	597	BC077801<br>NM_009455<br>X59863	4	4	4	4	4	12	13	8*		
xUBF mRNA for upstream binding factor	1911	X57201<br>X53390<br>AJ506039	22	22	30	22	58	48	78	53		
endoplasmic reticulum UDP-Glc/UDP-Gal transporter	1017	BC072878<br>BC011888<br>AY112732	2	5	17	3.5	14	12	65	13		
UDP-glucose ceramide glucosyltransferase	1182	BC084966<br>BC038711<br>BC077361	3	3	0	3	33	16	34	24.5*	0.25	+
Uroplakin 1A	627*	BC077369<br>NM_007000<br>BC042931	5	4	9	4.5	14	12	52	13		
Ubiquinol-cytochrome C reductase complex	1353	BC077311<br>NM_025899	15	11	19	13	29	29	51	29		

Vasodilator-stimulated phosphoprotein	1101	BC077932 BC072836 NM_003370	15	8	13	11.5	18	17	42	17.5			
Ventral anterior homeobox protein (Vax1)	792*	AF064601 AJ271730 XM_544036	10	4	23	7	18	4	64	11*			
Ventral anterior homeobox protein (Vax2+3)	873	AJ238649 AF113517 Y17791	4	6	6	5	10	9	28	9.5			
Von Hippel-Lindau binding protein 1	531	BC054252 BC092334 XM_420327	6	8	2	7	9	13	18	11		+	
Vg1 RNA binding protein	1779	AF064633 AF064634 NM_001006359	6	9	2	7.5	31	25	40	28	0.325		
Vimentin*	1368	X16843 X16844 X56134	17	13	27	15	16	25	65	20.5			
Tryptophanyl-tRNA synthetase	1425	BC046713 BC068695 XM_421368	15	13	26	14	37	22	58	29.5			
Wee1A kinase	1560*	U13962 AF035443 AK131218	14	24	29	19	48	35	103	41.5			
Wee1B, Wee1-like protein kinase	957	AF358869 AB071983 D30743	9	9	9	9	39	30	68	34.5			
Uterine sensitization-associated protein-1 (Wise-A)	639	AY255636 AY319928 overlap with BC078598 AY319926	9	5	11	7	17	8	24	12.5			
Xwnt-3	399*	M55054 L07538 NM_204675	2	3	0	2.5	15	8	25	11.5		+	
Wilms' tumor suppressor (WT1)	1131*	U42011 D82051 AB033633	7	3	8	5	22	27	55	24.5			
Cofilin (XAC)	504	U26270 U26269 NM_001004406	7	5	10	6	8	14	21	11			
XE2 (helix-loop-helix transcription factor E2)	363*	BC086475 U25961 NM_003199	3	1	2	2	7	4	14	5.5			
Xefiltin	1440	U63711 BC057721 XM_543999	12	31	24	21.5	26	42	82	34		+	+
Epidermis specific serine protease Prss27 (Xepsin)	1044	AB018694 BC083024 NM_031948	39	37	47	38	31	34	40	32.5			
Fork head related (XFD1)	1197	U65751 U65750 NM_204770	13	16	11	14.5	14	20	27	17			
Fork head protein (XFD2)	1074	X74315 X74316 XM_220287	29	12	37	20.5	18	30	53	24			+
Interleukin-1 beta-converting enzyme (Caspase 1)	1068*	D89783 D89785 NM_001223	31	43	68	37	23	21	44	22			
Xlmb (maternal B9.10 and B9.15 protein)	696	X73316 X73317 NM_017589	5	14	14	9.5	19	16	28	17.5			
L-myc oncogene (xL-myc)	1032	BC084925 L11363 NM_001033082	20	14	6	17	22	18	34	20		+	
Xnot (homeobox protein)	546	Z19577 L19566 NM_205354	5	11	3	8	6	15	22	10.5*		+	
TGF-beta related growth factor Xnr-4 (Xnr4)	1044*	AF410800 U79162 NM_018055	37	18	32	27.5	30	15	39	22.5			
XrnF12	1668*	AB114039 AB114040 XM_228541	18	16	24	17	43	35	48	39			
Xrpf (XrpFI beta 1) GA binding protein transcription factor	1155	BC046663 X91243 D13317	7	8	4	7.5	14	34	33	24*			
ZFTF (zinc finger transcription factor SLUG)	798	AF368041 AF368043 X77572	0	6	3	3	13	16	24	14.5			+
ZPB (zona pellucida glycoprotein)	1413	U44950 AB037700 AB025428	18	4	53	11	19	26	83	22.5			+
