# Supplementary material for: Multiple Mechanisms Promote the Retained Expression of Gene Duplicates in the Tetraploid Frog Xenopus laevis
Source: PLoS Genet. 2006 Apr 28;2(4):e56. doi: 10.1371/journal.pgen.0020056 (PMC1449897; doi:10.1371/journal.pgen.0020056)
Supplement: Table S3 — Results of test for different nonsynonymous substitution rates in each paralog (Model B versus C in Figure 3). (69 KB PDF) [file pgen.0020056.st003.pdf]

Supplementary Information Table 3. Results of test for different nonsynonymous substitution rates in each paralog. A three-rate model was compared to a 2-rate model (Models C and B in Fig. 3). Individual significance of the rate test is indicated with an asterisk at α = 0.05 and a P value of 1.0 is assigned if the ka/ks ratio (estimated by a separate test) of the paralog with the faster rate was lower than the other paralog and/or lower than the diploid lineage. Maximum likelihood estimates of *ka/ks* ratios are listed; these ratios are not necessarily equivalent to the ratio of the number of nonsynonymous and synonymous sites in Suppl. Info. Table 1 because those listed here consider multiple substitutions at each site. See text for further details and tablewide significance.

	Rate test						
Gene	ln(L) 2-rate	ln(L) 3-rate	$\chi 2$(df=1)	P value	*ka/ks* ratio α	*ka/ks* ratio β	*ka/ks* ratio diploid
Actin (skeletal, alpha 3)*	-1107.14	-1107.14	0.00	1.0000	0.0001	0.000	0.029
Activin Receptor-Like Kinase-2 (ALK-2)	-1558.96	-1558.93	0.05	1.0000	0.0835	0.102	0.028
Activin receptor II	-1687.15	-1686.74	0.83	0.3635	0.0701	0.155	0.028
Adipophilin - Adipose differentiation-	-1464.08	-1463.22	1.72	0.1903	0.2202	0.155	0.145
AE (Amidating Enzyme)	-3041.93	-3041.69	0.48	0.4888	0.0958	0.125	0.094
Albumin (serum)*	-2576.29	-2575.48	1.62	1.0000	0.3126	0.397	0.287
ALDH (Aldehyde dehydrogenase class1)	-1695.87	-1695.86	0.04	0.8463	0.2126	0.136	0.076
Alpha Globin	-567.48	-567.37	0.24	1.0000	0.0761	0.155	0.799
Amelogenin	-673.41	-671.70	3.41	0.0650	0.3746	0.590	0.367
Xenopus Anterior Neural Folds, Homeobox gene	-722.09	-722.08	0.01	0.9149	0.2289	0.176	0.139
Amyloid-Beta-like protein precursor	-2515.71	-2511.93	7.56	0.0060*	0.269	0.103	0.055
Apoptosis Inhibitor 5	-1545.94	-1545.52	0.84	0.3582	0.0453	0.074	0.022
AR (Androgen Receptor)	-485.22	-483.85	2.75	1.0000	0.0001	0.109	0.034
Liver L-arginase	-1220.90	-1203.75	34.30	0.0000*	0.0001	0.870	0.115
Arginase Type 2	-1111.95	-1111.13	1.65	0.1991	0.0435	0.099	0.011
Arrestin	-1228.15	-1227.86	0.58	0.4455	0.092	0.058	0.033
Aspartyl tRNA synthetase	-1653.61	-1653.35	0.53	0.4685	0.0629	0.031	0.026
Atonal Homolog 5	-508.64	-507.59	2.09	0.1482	0.4483	0.127	0.133
ATP synthase subunit B	-867.41	-867.35	0.13	0.7152	0.2928	0.101	0.085
Bambi (TGF-beta signalling)	-937.47	-935.41	4.12	1.0000	0.0797	0.131	0.144
Barren (brrn1, 13S condensin XCAP-H	-2417.75	-2417.75	0.01	1.0000	0.125	0.121	0.096
Bestrophin-2 (VMD2L1)	-2003.05	-2003.03	0.05	1.0000	0.3477	0.292	0.127
Beta Globin	-676.22	-675.85	0.75	1.0000	0.2977	0.431	1.068
Complement factor B (Bf B)	-3289.18	-3289.16	0.05	0.8282	0.4727	0.461	0.320
Biglycan	-1216.00	-1215.56	0.88	1.0000	0.0487	0.111	0.126
Bicaudal-C	-2057.11	-2056.48	1.26	0.2612	0.0988	0.092	0.041
Bridging integrator 1 (Amphiphysin II)	-1612.92	-1611.54	2.77	0.0963	0.2342	0.124	0.084
Bing4	-2028.20	-2025.84	4.72	0.0298*	0.1481	0.365	0.184
BMP (Bone Morphogenetic Protein)	-1548.64	-1548.35	0.59	1.0000	0.0458	0.058	0.061
Block of proliferation 1	-1170.45	-1169.89	1.12	0.2900	0.0739	0.289	0.089
Brachyury (T)	-1431.37	-1430.18	2.39	0.1224	0.0601	0.143	0.067
Serine/Threonine protein kinase	-2461.01	-2460.59	0.85	1.0000	0.093	0.085	0.064
Basic transcription element binding protein	-933.63	-933.08	1.09	1.0000	0.0848	0.079	0.210
B-cell translocation gene 1, anti-proliferative	-595.49	-595.26	0.46	0.4957	0.8109	0.203	0.059
Calcium homeostasis endoplasmic reticulum	-3079.75	-3075.21	9.07	0.0026*	0.206	0.093	0.042
Calnexin	-2220.62	-2220.25	0.75	0.3880	0.2773	0.120	0.171
Calponin H3 (clpH3)	-963.90	-963.88	0.03	1.0000	0.1365	0.063	0.056
Calreticulin	-1390.33	-1390.32	0.02	1.0000	0.0478	0.074	0.093
Carbonic anhydrase II	-990.91	-989.89	2.04	0.1533	0.495	0.161	0.185
Casein kinase I alpha S Casein kinase 1, alpha 1	-985.72	-985.02	1.40	0.2361	0.0001	0.014	0.000
CASK interacting protein 2	-4472.90	-4472.40	0.99	0.3194	0.2341	0.160	0.158
Procathepsin B	-1185.41	-1184.99	0.84	1.0000	0.0851	0.117	0.118
Beta Catenin interacting protein 1	-230.19	-229.50	1.39	0.2380	0.0001	0.192	0.000
Cystathionine-beta-synthase	-707.63	-707.56	0.13	0.7134	0.0581	0.039	0.039
voltage-dependent Calcium channel beta	-1570.68	-1569.47	2.43	0.1194	0.0755	0.163	0.062
CDC2 (cell division cycle 2, kinase)	-950.92	-949.35	3.14	0.0766	0.04	0.115	0.007

Cathepsin E	-1360.10	-1359.73	0.76	0.3841	0.1044	0.147	0.121
Carboxyl ester lipase	-1601.04	-1600.53	1.02	0.3128	0.1	0.066	0.053
Carboxyl ester lipase (bile salt-stimulated	-2007.85	-2007.67	0.38	1.0000	0.1469	0.112	0.105
Centrin	-514.75	-514.58	0.35	1.0000	0.0407	0.030	0.074
Cerebellin 2 precursor protein	-774.15	-773.86	0.59	0.4411	0.2746	0.326	0.071
Complement factor I (C3b/C4b inactivator)	-2444.96	-2443.89	2.15	0.1425	0.3955	0.243	0.278
Cystic fibrosis transmembrane	-4819.42	-4819.39	0.06	0.8141	0.1428	0.196	0.090
Cortical granule lectin	-1255.47	-1254.36	2.22	1.0000	0.1673	0.179	0.208
Choroideremia (Rab escort protein 1)	-2445.10	-2442.40	5.40	0.0201*	0.1792	0.367	0.326
Carbohydrate sulfotransferase 11	-1072.50	-1071.89	1.21	0.2705	0.0977	0.069	0.056
Cell death-inducing DFFA-like effector c	-872.78	-872.78	0.01	0.9278	0.2069	0.128	0.149
C-Jun (c-jun proto oncogene)	-1110.08	-1110.04	0.08	1.0000	1.1428	0.210	0.101
Dipeptidase 2 (metallopeptidase M20	-1608.81	-1608.34	0.95	0.3294	0.1719	0.110	0.062
alpha-1 Collagen type II	-4303.92	-4302.15	3.53	0.0604	0.1642	0.227	0.131
Connexin 31 (Gap junction beta-3 protein)	-921.02	-920.68	0.67	0.4129	0.1879	0.140	0.042
Contactin/F3/F11 (Contactin A)	-3600.35	-3600.33	0.04	0.8422	0.1801	0.183	0.096
Coronin	-1686.76	-1686.76	0.00	0.9977	0.1536	0.129	0.103
Cortactin	-1867.07	-1866.91	0.32	0.5717	0.1061	0.097	0.103
Cytoplasmic polyadenylation element	-1815.61	-1814.67	1.87	0.1712	0.1266	0.057	0.036
CRY2 (cryptochrome 2)	-1847.24	-1847.20	0.10	0.7555	0.1192	0.088	0.069
Crystallin, beta A1	-765.68	-764.92	1.54	0.2153	0.3642	0.276	0.136
Cathepsin S (CTSS)	-1299.16	-1299.16	0.01	1.0000	0.3478	0.237	0.183
Cullin3 (Cul3)	-2234.99	-2234.82	0.34	0.5618	0.0055	0.010	0.000
CyclinE	-1503.61	-1503.18	0.86	0.3527	0.2048	0.136	0.149
Brain Dopamine receptor D2	-1175.53	-1175.15	0.77	0.3816	0.1149	0.210	0.099
Dapper 1, antagonist of beta-catenin	-3004.40	-3002.70	3.39	0.0655	0.1879	0.194	0.105
Death-associated protein kinase 1	-4610.16	-4608.11	4.11	0.0427*	0.0825	0.125	0.040
Drebrin-like	-1407.14	-1407.12	0.04	0.8408	0.2466	0.388	0.142
Debranching enzyme homolog 1	-1899.47	-1897.85	3.24	0.0719	0.1815	0.133	0.084
Deleted in colorectal cancer tumor	-621.87	-619.61	4.51	0.0338*	0.2898	1.029	0.145
Desmin	-1519.07	-1518.86	0.43	0.5127	0.1035	0.185	0.035
Hand2	-408.75	-407.34	2.81	0.0934	0.0935	0.126	0.000
Cytoplasmic dynein light-intermediate chain 1	-1662.79	-1662.78	0.02	0.8756	0.1075	0.117	0.063
Dipeptidylpeptidase 3	-2697.45	-2697.43	0.04	1.0000	0.1638	0.271	0.149
Dullard	-713.62	-712.24	2.77	0.0959	0.0001	0.112	0.011
Dystroglycan (DAG1)	-3002.76	-3002.58	0.35	1.0000	0.1178	0.106	0.182
Dystrophin	-1764.46	-1761.69	5.54	0.0186*	0.1282	0.000	0.028
Helix-loop-helix transcription factor XE1	-505.15	-504.74	0.83	0.3612	0.1279	0.193	0.066
E2 (transcription factor E2)	-2137.36	-2133.77	7.18	0.0074*	0.1941	0.102	0.094
met-mesencephalon-olfactory transcription	-1839.06	-1838.73	0.66	0.4156	0.1421	0.1624	0.011
CCAAT/enhancer binding protein (C/EBP), alpha	-1114.55	-1113.12	2.86	0.0910	0.1394	0.205	0.156
Endothelin receptor type A	-1430.42	-1429.55	1.75	0.1861	0.208	0.060	0.098
EF (Elongation Factor-1 alpha, 42Sp48)	-1376.60	-1375.17	2.86	0.0906	0.018	0.071	0.011
Aurora kinase A (EG2)	-1494.96	-1494.70	0.52	1.0000	0.0793	0.127	0.138
Engrailed 2 (EN2)	-916.80	-915.64	2.31	1.0000	0.0605	0.151	0.228
Enkephalin A (proenkephalin A)*	-743.61	-743.20	0.82	0.3664	0.2353	0.090	0.067
ENO (alpha enolase) (2-phosphoglycerate	-1424.27	-1422.29	3.97	1.0000	0.0001	0.065	0.140
Era (Estrogen Receptor alpha)	-1374.29	-1372.84	2.91	0.0881	0.0558	0.077	0.066
Enhancer of split groucho	-2263.09	-2260.69	4.80	0.0285*	0.0001	0.056	0.033
Enhancer of zeste	-2401.48	-2399.14	4.68	0.0306*	0.065	0.093	0.010
Focal adhesion kinase	-3396.32	-3394.95	2.75	0.0971	0.052	0.087	0.042

Transcription factor (clone XLFB1)	-351.23	-351.18	0.11	1.0000	0.377	0.222	0.086
XFD-4	-1561.18	-1561.03	0.29	1.0000	0.2515	0.116	0.046
Flap endonuclease-1	-1280.68	-1280.52	0.32	0.5725	0.0928	0.054	0.076
FetuinB	-2022.10	-2021.38	1.42	1.0000	0.354	0.341	0.515
Ftz-F1-related orphan receptor (xFF1r)	-1433.94	-1432.01	3.87	0.0491*	0.1088	0.027	0.017
FGF (embryonic fibroblast growth factor	-687.79	-687.62	0.33	1.0000	0.1861	0.478	0.295
Fibroblast growth factor receptor	-2730.49	-2728.46	4.04	0.0443*	0.0976	0.173	0.048
Fibrinogen alpha	-2850.21	-2847.19	6.03	0.0141*	0.1593	0.409	0.215
Flotillin	-1417.32	-1417.29	0.07	1.0000	0.208	0.172	0.085
fms-related tyrosine kinase 1/vascular	-2823.81	-2823.65	0.31	0.5781	0.4305	0.278	0.231
Fms-interacting protein (NF2/meningioma	-2321.61	-2321.04	1.15	0.2837	0.1932	0.119	0.049
alpha-fodrin (Xen alpha 1)	-791.13	-791.09	0.08	0.7796	0.0855	0.201	0.063
c-fos proto-oncogene	-296.58	-295.92	1.32	0.2506	0.2441	0.376	0.202
Succinate dehydrogenase	-2235.14	-2234.75	0.78	0.3767	0.1683	0.093	0.090
Frequenin	-569.42	-569.41	0.04	0.8483	0.0175	0.019	0.000
Fascin	-1647.17	-1646.23	1.87	1.0000	0.091	0.091	0.061
Furin*	-1984.40	-1982.85	3.10	0.0783	0.0666	0.130	0.082
Fused toes homolog	-946.42	-945.74	1.36	0.2441	0.0825	0.143	0.065
FYN (c-fyn, Fyn proto-oncogene)	-1609.57	-1609.57	0.00	1.0000	0.027	0.035	0.011
Galectin	-1064.71	-1064.54	0.34	0.5602	0.2548	0.261	0.163
alpha subunit of Gq Gtp-binding protein	-1100.02	-1099.50	1.05	1.0000	0.0643	0.023	0.080
GATA-binding protein transcription factor	-1375.95	-1375.85	0.18	1.0000	0.3929	0.229	0.169
Transcription factor xGata5	-1297.08	-1296.31	1.54	0.2140	0.124	0.077	0.056
Growth hormone A	-485.58	-483.57	4.01	0.0452*	0.0501	0.268	0.054
Guanylate kinase 1	-732.88	-732.16	1.44	0.2308	0.4128	0.121	0.119
Glycogenin 1 (mitotic phosphoprotein	-1115.05	-1114.90	0.31	0.5788	0.1308	0.160	0.109
Holocytochrome c synthase (cytochrome c	-1200.74	-1197.78	5.93	0.0149*	0.1613	0.705	0.215
cephalic Hedgehog, sonic hedgehog protein	-1444.97	-1444.67	0.61	0.4353	0.2256	0.237	0.170
Transcription factor XHEN1	-427.70	-427.58	0.25	0.6185	0.1222	0.145	0.079
Hypoxia-inducible factor 1 alpha	-1628.67	-1627.60	2.14	0.1438	0.1676	0.123	0.047
SafA - scaffold attachment factor A	-2804.69	-2803.44	2.49	0.1145	0.1648	0.122	0.090
Homeobox 2/2.3*	-547.22	-547.01	0.41	1.0000	0.102	0.209	0.074
Insulin*	-355.49	-354.98	1.03	0.3107	0.2341	0.303	0.037
Integrin beta-1 subunit*	-2559.93	-2559.93	0.01	1.0000	0.0406	0.033	0.051
Inversin	-4147.08	-4146.88	0.40	0.5277	0.3213	0.240	0.195
Ubiquitin carboxyl-terminal hydrolase 5	-2813.14	-2813.09	0.09	0.7679	0.1411	0.097	0.056
Kf-1 protein (Adgr34)	-2413.41	-2413.40	0.03	1.0000	0.1799	0.132	0.083
Kit receptor tyrosine kinase (c-kit)	-3577.21	-3577.20	0.02	1.0000	0.2273	0.354	0.165
Kinesin-like protein 2	-3095.42	-3095.37	0.10	0.7520	0.1627	0.132	0.130
L1 (ribosomal protein L1)	-1188.41	-1187.34	2.15	0.1425	0.0901	0.012	0.041
L14 (ribosomal protein L14)	-622.20	-622.20	0.00	1.0000	0.1338	0.128	0.154
Lamin B	-2056.23	-2056.12	0.21	1.0000	0.151	0.138	0.128
Lamina associated polypeptide 2	-1995.50	-1994.88	1.22	1.0000	0.2711	0.292	0.331
Clathrin, light polypeptide (Lcb)	-679.10	-679.06	0.08	0.7841	0.1609	0.115	0.035
Lactate dehydrogenase	-1194.51	-1194.30	0.41	1.0000	0.1167	0.166	0.219
LEF-1 (lymphoid enhancer factor)	-1179.90	-1179.87	0.07	0.7913	0.1265	0.156	0.020
TGF-beta family member Lefty-A	-1267.13	-1266.99	0.28	0.5978	0.1193	0.102	0.114
LIM domain binding protein	-1103.34	-1102.66	1.37	0.2419	0.0001	0.018	0.000
Lipocalin (Ptgds)	-562.33	-561.87	0.92	0.3366	0.2753	0.129	0.132
Lpa1R (lysophosphatidic acid receptor)	-1094.97	-1094.63	0.69	0.4077	0.0803	0.043	0.000
LR (Leptin Receptor)	-387.45	-387.35	0.20	0.6536	0.1344	0.128	0.026

Lipoprotein (LDL) receptor-related protein	-663.39	-663.39	0.00	0.9768	0.0294	0.039	0.028
Autoantigen La (La protein)	-1612.49	-1612.46	0.08	1.0000	0.1291	0.162	0.179
Microfibrillar-associated protein 1	-1361.84	-1361.83	0.01	0.9163	0.0726	0.064	0.031
Myristoylated alanine-rich C kinase substrate	-715.64	-714.24	2.80	0.0945	0.681	0.241	0.247
XMax2 and XMax4	-355.61	-354.92	1.38	1.0000	0.0594	0.000	0.106
Myogenin	-743.15	-742.95	0.40	0.5295	0.1179	0.147	0.021
Myozenin1	-1153.56	-1152.70	1.71	0.1904	0.3979	0.197	0.175
N-CAM (neural cell adhesion molecule)*	-3840.77	-3837.77	6.00	0.0143*	0.3101	0.201	0.226
NF-M1 (middle molecular	-2902.80	-2902.75	0.09	0.7667	0.201	0.217	0.130
neurogenin-related 1 (X-NGNR-1)	-816.39	-816.19	0.41	1.0000	0.1907	0.173	0.178
Internexin neuronal intermediate filament	-1577.68	-1576.87	1.62	1.0000	0.1362	0.144	0.059
NK3 transcription factor related, locus 1	-906.58	-906.55	0.06	0.8105	0.4125	0.337	0.240
Nonmuscle myosin II heavy chain A	-2838.50	-2838.30	0.40	0.5285	0.0866	0.059	0.059
Nonmuscle myosin heavy chain B	-1057.96	-1054.81	6.29	0.0121*	0.1306	0.012	0.036
Nucleolar-localized protein NO38	-1095.93	-1093.39	5.08	0.0242*	0.0822	0.256	0.145
Nucleobindin 1	-617.99	-617.65	0.68	1.0000	0.0545	0.051	0.025
Nucleoplasmin	-668.03	-667.86	0.34	1.0000	0.1155	0.140	0.205
Nucleoporin (Nup88)	-2630.12	-2628.34	3.56	0.0591	0.0973	0.203	0.140
OLPA (Dorphin)	-956.32	-956.12	0.40	0.5265	0.2879	0.473	0.111
Olfactory marker protein (XOMP)	-630.77	-630.69	0.16	1.0000	0.1278	0.378	0.122
OncogenesC-ets-1 (c-ets-1b proto-	-1363.14	-1363.09	0.11	1.0000	0.0418	0.027	0.042
OncogenesC-ets-2 (ets-2a proto-oncogene)*	-1607.82	-1607.81	0.03	0.8681	0.126	0.080	0.042
OncogenesC-myc (myelocytomatosis	-1407.29	-1407.24	0.09	0.7596	0.094	0.171	0.053
Dynactin 2 (p50)	-1303.85	-1303.76	0.19	1.0000	0.0654	0.080	0.090
PACSIN2	-1232.49	-1230.91	3.14	0.0762	0.148	0.090	0.042
Convertase PC2	-1942.75	-1942.33	0.84	1.0000	0.0778	0.072	0.012
Prolyl isomerase (Pin1)	-525.01	-525.00	0.02	0.8849	0.08	0.057	0.080
PKC (protein kinase C,delta)	-2348.88	-2348.75	0.24	0.6219	0.1042	0.163	0.069
Plakoglobin	-2377.19	-2377.19	0.00	1.0000	0.0709	0.058	0.047
Peripheral myelin protein 22	-524.87	-524.19	1.36	0.2444	0.143	0.055	0.113
POMC (pro-opiomelanocortin)*	-936.16	-936.16	0.01	1.0000	0.2699	0.105	0.048
POU domain Gene 1	-1122.05	-1118.60	6.90	0.0086*	0.1843	0.045	0.019
POU3	-905.52	-902.93	5.18	0.0228*	0.222	0.027	0.091
Phosphorylase phosphatase (Ppp2B)	-1730.29	-1727.74	5.08	0.0241*	0.1012	0.007	0.005
Protein phosphatase 4, regulatory subunit 2	-1547.25	-1547.01	0.48	1.0000	0.3325	0.203	0.177
LIM protein Prickle	-2830.32	-2829.73	1.19	0.2744	0.1851	0.095	0.076
Prolactin Receptor	-2450.50	-2450.05	0.90	1.0000	0.3075	0.273	0.183
Prothymosin	-317.57	-317.53	0.08	0.7720	0.2172	0.165	0.032
Phosphorylase, glycogen; brain	-2761.33	-2761.27	0.13	0.7173	0.1083	0.054	0.041
RAB18 (member RAS oncogene family)	-649.70	-649.19	1.02	0.3122	0.0466	0.130	0.033
Rac GTPase	-570.80	-570.62	0.36	0.5506	0.0361	0.056	0.000
Rad51	-1008.02	-1007.68	0.69	0.4077	0.0425	0.019	0.018
Rag-1	-1298.27	-1298.15	0.25	0.6189	0.0902	0.156	0.066
Ral interacting protein (rlip gene) RalA	-1925.97	-1922.94	6.06	0.0138*	0.0363	0.105	0.059
RalB	-596.95	-596.62	0.67	1.0000	0.0227	0.000	0.024
Retinoic acid receptor alpha	-1332.57	-1328.42	8.30	0.0040*	0.0328	0.317	0.029
RDS35 (retinal degradation	-1258.75	-1256.33	4.84	0.0278*	0.1647	0.213	0.106
rds/peripherin (rds38)	-1139.23	-1133.82	10.82	0.0010*	0.3136	0.042	0.045
Requiem	-1265.44	-1265.41	0.06	1.0000	0.1896	0.094	0.030
Rhodopsin	-1138.75	-1138.13	1.24	0.2648	0.0455	0.475	0.174
Ringo (p33 ringo, ls26) (rapid inducer of G 2/M	-1135.21	-1134.10	2.23	1.0000	0.1923	0.368	0.150

RIO kinase 2	-2055.42	-2055.40	0.04	0.8403	0.1941	0.201	0.116
Rwdd1 (RWD domain containing 1)	-863.37	-862.67	1.40	0.2370	0.2657	0.133	0.084
Retinal homeobox A	-1075.35	-1074.97	0.75	0.3849	0.0703	0.114	0.080
Rxrb (retinoid X receptor beta)	-1399.48	-1399.17	0.62	0.4306	0.0581	0.125	0.072
Sister chromatid cohesion establishment	-856.35	-856.30	0.10	1.0000	0.1417	0.214	0.151
syndecan 2 (heparan sulfate proteoglycan 1,	-626.00	-625.95	0.10	1.0000	0.1871	0.278	0.063
Sek-1 receptor tyrosine kinase	-3004.62	-3004.59	0.07	1.0000	0.063	0.048	0.005
Selenoprotein I	-1370.88	-1370.18	1.39	1.0000	0.2496	0.178	0.067
Selenoprotein T	-442.66	-442.58	0.15	1.0000	0.1943	0.120	0.000
Septin 11	-1010.26	-1010.26	0.00	1.0000	0.0516	0.067	0.054
septin A (XlSeptA)	-1096.18	-1096.18	0.00	1.0000	0.0781	0.073	0.000
serum/glucocorticoid regulated kinase	-1376.08	-1369.08	14.00	0.0002*	0.0102	0.143	0.050
Shab12 (delayed rectifier potassium ion	-565.96	-565.87	0.18	0.6756	0.0761	0.058	0.017
Siah-interacting protein	-811.15	-810.14	2.02	0.1555	0.3826	0.230	0.092
Sloan-Kettering viral oncogene homolog	-2307.30	-2307.27	0.07	1.0000	0.128	0.154	0.050
Histone stem-loop binding protein (SLBP)	-987.69	-987.14	1.08	1.0000	0.2134	0.121	0.271
suc1-associated neurotrophic factor	-1757.22	-1757.20	0.05	0.8283	0.1378	0.242	0.094
Sox11 (XLS13)	-1242.28	-1242.11	0.34	0.5600	0.0678	0.196	0.059
Sox17a (HMG box transcription factor	-1376.31	-1373.93	4.77	0.0290*	0.1106	0.245	0.132
Sox18 (Transcription factor SOX-18)	-1103.03	-1102.73	0.60	1.0000	0.1917	0.131	0.085
SP22	-549.87	-549.77	0.20	1.0000	0.0465	0.082	0.171
Sparc	-1017.59	-1015.41	4.35	0.0370*	0.2467	0.101	0.044
Spats2 (spermatogenesis	-1964.55	-1962.01	5.08	0.0242*	0.4294	0.100	0.160
Spermatid perinuclear RNA binding protein	-1633.67	-1631.70	3.93	0.0474*	0.297	0.213	0.116
Sprouty-2	-1055.90	-1055.30	1.19	0.2745	0.2637	0.204	0.132
Sulfide quinone reductase-like (yeast)	-1522.53	-1522.47	0.10	0.7464	0.1095	0.177	0.060
Src (pp60c-src protein)	-1645.55	-1645.50	0.09	1.0000	0.057	0.066	0.013
Stanniocalcin 1	-881.57	-880.69	1.76	0.1847	0.1693	0.108	0.070
Staufen 1	-1450.62	-1450.60	0.06	0.8138	0.1882	0.161	0.100
Stress-induced-phosphoprotein 1	-1837.99	-1837.77	0.44	1.0000	0.1874	0.130	0.061
Stomatin	-455.91	-455.56	0.68	0.4090	0.1148	0.030	0.046
Strabismus	-1558.21	-1554.05	8.33	0.0039*	0.0455	0.000	0.005
SUG1	-1166.75	-1166.58	0.34	0.5597	0.0156	0.011	0.007
translation initiation factor SUI1	-319.22	-319.22	0.00	1.0000	0.0001	0.000	0.000
Sumo	-302.70	-300.61	4.19	0.0407*	0.2404	0.000	0.000
Survivin (Xsvv1)	-548.98	-548.91	0.14	1.0000	0.1112	0.133	0.136
Synaptobrevin	-349.33	-347.27	4.13	0.0422*	0.2793	0.000	0.060
Synaptophysin	-1011.08	-1010.96	0.23	0.6344	0.2607	0.144	0.076
Xwnt8 inhibitor sizzled (szl)	-970.36	-969.81	1.10	1.0000	0.1416	0.135	0.154
TAF-Ibeta	-795.51	-795.51	0.00	0.9956	0.0464	0.054	0.033
T-box transcription factor Tbx5	-365.56	-365.56	0.01	0.9145	0.0548	0.034	0.000
TCRzeta subunit	-656.30	-655.90	0.79	1.0000	0.2344	0.225	0.258
Bax Inhibitor-1, testis enhanced gene	-779.13	-779.03	0.21	0.6462	0.3343	0.179	0.034
TRK-fused protein TFG	-1256.21	-1256.19	0.06	1.0000	0.1152	0.108	0.109
Thyroid Hormone Receptor alpha*	-1309.97	-1308.31	3.32	0.0686	0.0517	0.110	0.043
Thyroid Hormone Receptor beta*	-1137.02	-1136.78	0.48	0.4881	0.0654	0.101	0.022
Mesoderm Posterior (Mesp)	-1190.88	-1190.84	0.09	1.0000	0.2265	0.267	0.224
cytotoxic granule-associated RNA binding	-1215.41	-1214.22	2.38	0.1229	0.0738	0.033	0.016
TIAR	-1259.56	-1258.98	1.17	0.2802	0.0523	0.174	0.026
Tyrosine kinase	-508.30	-508.10	0.39	0.5338	0.0756	0.324	0.000
IGF (Insulin-like Growth Factor) Receptor	-483.55	-483.03	1.05	0.3056	0.0507	0.145	0.033

Transducer of erbB	-939.50	-936.92	5.16	0.0231*	0.0319	0.119	0.079
Transferrin	-2744.61	-2742.98	3.25	0.0714	0.2793	0.244	0.236
Thyrotropin-releasing Hormone	-1001.41	-1001.15	0.51	0.4731	0.6228	0.509	0.293
Thyrotropin-releasing Hormone Receptor 1	-1271.59	-1270.76	1.67	0.1961	0.139	0.060	0.044
Neurotrophin receptor B xTrkB-alpha	-1704.67	-1704.04	1.26	0.2620	0.3707	0.169	0.181
fast skeletal Troponin C	-447.63	-446.93	1.39	1.0000	0.025	0.000	0.040
unitary non-NMDA glutamate receptor	-1625.32	-1625.29	0.06	0.8108	0.1173	0.106	0.080
Ubiquiton-conjugating enzyme e2e	-647.60	-647.60	0.00	0.9881	0.3764	0.108	0.099
xUBF mRNA for upstream binding factor	-2230.17	-2230.17	0.01	0.9227	0.0889	0.100	0.087
endoplasmic reticulum UDP-Glc/UDP-Gal	-1107.87	-1107.63	0.48	0.4895	0.0561	0.139	0.083
UDP-glucose ceramide glucosyltransferase	-1212.71	-1212.70	0.03	0.8731	0.0324	0.073	0.000
Uroplakin 1A	-716.72	-716.67	0.09	0.7593	0.095	0.075	0.054
Ubiquinol-cytochrome C reductase complex,	-1530.74	-1530.64	0.19	0.6647	0.202	0.127	0.116
Vasodilator-stimulated phosphoprotein	-1235.12	-1233.74	2.76	0.0968	0.3691	0.181	0.125
Ventral anterior homeobox protein	-975.47	-974.31	2.31	1.0000	0.1796	0.231	0.088
Ventral anterior homeobox protein	-912.94	-912.73	0.43	0.5125	0.1763	0.287	0.102
Von Hippel-Lindau binding protein 1	-603.05	-602.90	0.30	0.5818	0.1759	0.181	0.027
Vg1 RNA binding protein	-1805.61	-1805.29	0.65	0.4216	0.055	0.105	0.018
Vimentin*	-1625.30	-1624.60	1.40	0.2366	0.3532	0.136	0.120
Tryptophanyl-tRNA synthetase	-1700.97	-1700.92	0.09	1.0000	0.1188	0.169	0.128
Wee1A kinase	-1913.25	-1912.26	1.99	0.1586	0.0771	0.228	0.077
Wee1B, Wee1-like protein kinase	-1089.34	-1089.34	0.00	1.0000	0.08	0.066	0.032
Uterine sensitization-associated protein-1	-766.25	-765.82	0.85	1.0000	0.1458	0.173	0.138
Xwnt-3	-415.24	-415.13	0.21	0.6454	0.0341	0.115	0.000
Wilms' tumor suppressor (WT1)	-1209.61	-1209.45	0.32	0.5730	0.0995	0.036	0.037
Cofilin (XAC)	-595.44	-595.26	0.37	0.5422	0.2643	0.0812	0.125
XE2 (helix-loop-helix transcription factor E2)	-362.66	-362.13	1.05	0.3049	0.1487	0.1117	0.045
Xefiltin	-1745.75	-1742.77	5.97	0.0146*	0.1503	0.1932	0.070
Epidermis specific serine protease Prss27	-1622.08	-1622.08	0.00	1.0000	0.4139	0.3615	0.468
Fork head related (XFD1)	-1398.08	-1397.93	0.30	1.0000	0.257	0.2408	0.123
Fork head protein (XFD2)	-1445.08	-1441.53	7.11	0.0077*	0.3813	0.1054	0.176
Interleukin-1 beta-converting enzyme	-1733.46	-1733.32	0.27	0.6020	0.4472	0.6068	0.526
Xlmb (maternal B9.10 and B9.15 protein)	-879.69	-878.73	1.91	0.1668	0.0777	0.2514	0.109
L-myc oncogene (xL-myc)	-1225.21	-1224.63	1.16	0.2809	0.2824	0.255	0.060
Xnot (homeobox protein)	-648.13	-646.91	2.44	1.0000	0.2276	0.223	0.031
TGF-beta related growth factor Xnr-4 (Xnr4)	-1500.96	-1498.69	4.53	0.0334*	0.3928	0.3794	0.269
XrnF12	-1907.36	-1907.36	0.00	1.0000	0.1525	0.1535	0.188
Xrpf (XrpFI beta 1) GA binding protein	-1212.90	-1212.86	0.08	1.0000	0.1375	0.0688	0.034
ZFTF (zinc finger transcription factor	-821.66	-817.49	8.35	0.0039*	0.0001	0.1058	0.034
ZPB (zona pellucida glycoprotein)	-1776.40	-1773.26	6.27	0.0123*	0.4215	0.05	0.216
