# Supplementary material for: Multiple Mechanisms Promote the Retained Expression of Gene Duplicates in the Tetraploid Frog Xenopus laevis
Source: PLoS Genet. 2006 Apr 28;2(4):e56. doi: 10.1371/journal.pgen.0020056 (PMC1449897; doi:10.1371/journal.pgen.0020056)
Supplement: Table S4 — Tests for complementary patterns of substitution using the paralog heterogeneity test and runs test for dichotomous variables on nonsynonymous and synonymous substitutions. (52 KB PDF) [file pgen.0020056.st004.pdf]

Supplementary Information Table 4. Tests for complementary patterns of substitution using the paralog heterogeneity test and runs test for dichotomous variables on nonsynonymous and synonymous substitutions. P values of paralog heterogenety tests are reported for two (P2) or three domains (P3). Tests were not conducted if there were a small number of substitutions in one paralog (see text); individually significant values (P < 0.05) are indicated with an asterisk.

	nonsynonymous substitutions			synonymous substitutions		
	paralog heterogeneity test		Runs test	paralog heterogeneity test		Runs test
Gene name	P2	P3		P2	P3	
Actin (skeletal, alpha 3)*	-	-	-	0.7400	0.1220	0.0789
Activin Receptor-Like Kinase-2 (ALK-2)	0.1000	0.0920	0.4093	0.9950	0.9860	0.6453
Activin receptor II	0.9820	0.5800	0.7317	0.9900	0.9770	0.2607
Adipophilin (fatvg)	0.8370	0.9420	0.4892	0.9040	0.0980	0.3024
AE (Amidating Enzyme)	0.4300	0.0150*	0.3314	0.6140	0.2600	0.0325*
Albumin (serum)*	0.0670	0.1480	0.0978	0.0340*	0.0270*	0.0030*
ALDH (Aldehyde dehydrogenase class1)	0.4040	0.5930	0.0245*	0.9310	0.9280	0.7483
Alpha Globin	0.0580	0.1290	0.3911	0.1130	0.1110	0.9449
Amelogenin	0.9460	0.9490	0.7877	0.3210	0.5720	0.6611
Xenopus Anterior Neural Folds, Homeobox gene	0.6340	0.2430	0.8382	0.5055	0.5850	0.1097
Amyloid-Beta-like protein precursor	0.1890	0.0690	0.7964	0.9470	0.8260	0.8311
Apoptosis Inhibitor 5	0.5590	0.7620	0.5438	0.0680	0.0000*	0.2401
AR (Androgen Receptor)	-	-	-	-	-	-
Liver L-arginase	-	-	-	-	-	-
Arginase Type 2	0.9605	0.3690	0.9160	0.0420*	0.1330	0.0263*
Arrestin	0.8600	0.9520	0.0744	0.2410	0.3350	0.5997
Aspartyl tRNA synthetase	0.0390*	0.0500	0.7193	0.8010	0.7460	0.6021
Atonal Homolog 5	0.4560	0.6530	0.9517	0.3490	0.4740	0.2661
ATP synthase subunit B	0.2150	0.3730	0.8601	0.4210	0.4670	0.6387
Bambi (TGF-beta signalling)	0.1640	0.1990	0.2208	0.1110	0.4840	0.7151
Barren (brrn1, 13S condensin XCAP-H subunit)	0.2580	0.1580	0.4487	0.6180	0.7850	0.5241
Bestrophin-2 (VMD2L1)	0.6130	0.3680	0.6110	0.3750	0.5800	0.0398*
Beta Globin	0.8320	0.2550	0.6675	0.2020	0.3870	0.0539
Complement factor B (Bf B) (MHC class III gene)	0.1060	0.2570	0.0263*	0.7600	0.3280	0.7251
Biglycan	0.8110	0.8570	0.2370	0.7615	0.9020	0.9060
Bicaudal-C	0.0990	0.1840	0.4466	0.0260*	0.0580	0.2421
Bridging integrator 1 (Amphiphysin II)	0.4140	0.0590	0.0489*	0.2790	0.1000	0.0159*
Bing4	0.5030	0.3060	0.8368	0.8370	0.7830	0.3028
BMP (Bone Morphogenetic Protein) receptor	0.7950	0.4630	0.0825	0.9640	0.9340	0.5981
Block of proliferation 1	0.8470	0.9680	0.4702	0.9220	0.9720	0.5475
Brachyury (T)	0.7340	0.8290	0.8311	0.1200	0.2140	0.9680
Serine/Threonine protein kinase (c-RMIL)	0.3610	0.3880	0.0789	0.0970	0.3110	0.3378
Basic transcription element binding protein	-	-	-	0.1560	0.0730	0.5217
B-cell translocation gene 1, anti-proliferative	0.0020*	0.0070*	0.2248	0.9360	0.9760	0.4624
Calcium homeostasis endoplasmic reticulum protein	0.3930	0.6020	0.5054	0.9830	0.9280	0.5813
Calnexin	0.1270	0.0410*	0.1412	0.1050	0.3100	0.4393
Calponin H3 (clpH3)	0.6890	0.1180	0.8006	0.5410	0.3940	0.0332*
Calreticulin	0.8660	0.6940	0.5890	0.0820	0.1230	0.3719
Carbonic anhydrase II	0.7540	0.8250	0.8223	0.1570	0.2430	0.2826
Casein kinase I alpha S (Csnk1a1)	-	-	-	0.6480	0.8260	0.3338
CASK interacting protein 2	0.7150	0.3970	0.6820	0.8890	0.9810	0.3709
Procathepsin B	0.2950	0.5270	0.1503	0.6160	0.8300	0.8752
Beta Catenin interacting protein 1 (catnbip1)	-	-	-	-	-	-
Cystathionine-beta-synthase	0.9055	0.9740	0.8859	0.3290	0.2900	0.0220*
voltage-dependent Calcium channel beta subunit	0.2210	0.3700	0.7704	0.0030*	0.0010*	0.0016*
CDC2 (cell division cycle 2, kinase)	0.7550	0.7275	0.6180	0.4510	0.6670	0.8004

Cathepsin E	0.7390	0.8230	0.2405	0.6390	0.8170	0.6720
Carboxyl ester lipase	0.4060	0.3060	0.1230	0.9860	0.9820	0.4987
Carboxyl ester lipase (bile salt-stimulated lipase)	0.2600	0.4690	0.1369	0.1220	0.2550	0.6596
Centrin	-	-	-	0.0280*	0.0640	0.7910
Cerebellin 2 precursor protein	0.9960	0.4440	0.2897	0.7420	0.8510	0.5142
Complement factor I (C3b/C4b inactivator)	0.5450	0.1890	0.6225	0.1150	0.3070	0.9191
Cystic fibrosis transmembrane conductance regulator	0.2380	0.1230	0.0391*	0.9970	0.2110	0.7162
Cortical granule lectin	0.9030	0.0590	0.6410	0.1875	0.0600	0.9516
Choroideremia (Rab escort protein 1)	0.9690	0.5580	0.3615	0.9080	0.8290	0.3702
Carbohydrate sulfotransferase 11 (Chst11)	0.0110*	0.0050*	0.0881	0.0850	0.1210	0.1118
Cell death-inducing DFFA-like effector c (CIDE-3alpha)	0.8900	0.0860	0.1297	0.3970	0.4850	0.6535
C-Jun proto-oncogene (AP-1, Activator Protein)	0.5330	0.3180	0.2779	0.8340	0.8780	0.8821
Dipeptidase 2 (metallopeptidase M20 family)	0.0820	0.0680	0.4265	0.9720	0.6230	0.2856
alpha-1 Collagen type II	0.4680	0.6020	0.2774	0.7740	0.4640	0.3927
Connexin 31 (Gap junction beta-3 protein)	0.4560	0.7090	0.5085	0.3150	0.5610	0.1115
Contactin/F3/F11 (Contactin A)	0.6620	0.6330	0.2701	0.4030	0.7100	0.3679
Coronin	0.6960	0.7560	0.1042	0.1840	0.0840	0.6929
Cortactin	0.3380	0.2270	0.1984	0.3250	0.2430	0.2439
Cytoplasmic polyadenylation element binding protein	0.5150	0.5510	0.1509	0.0810	0.1160	0.5035
CRY2 (cryptochrome 2)	0.6050	0.7140	0.9125	0.8280	0.3240	0.3694
Crystallin, beta A1	0.9790	0.2740	0.7393	0.0470*	0.1710	0.3194
Cathepsin S (CTSS)	0.2250	0.3150	0.3667	0.9040	0.9180	0.5787
Cullin3 (Cul3)	-	-	-	0.9600	0.9800	0.1359
CyclinE	0.7740	0.8660	0.4828	0.2905	0.5040	0.9573
Brain Dopamine receptor D2	0.1905	0.3920	0.9106	0.7660	0.9070	0.6155
Dapper 1, antagonist of beta-catenin (Frodo)	0.1110	0.2790	0.0330*	0.1670	0.3930	0.1575
Death-associated protein kinase 1	0.1160	0.2520	0.7031	0.1680	0.2480	0.6978
Drebrin-like	0.5960	0.3390	0.7424	0.4060	0.7530	0.7693
Debranching enzyme homolog 1	0.0840	0.0230*	0.1703	0.2750	0.3600	0.6467
Deleted in colorectal cancer tumor suppressor	0.7980	0.8880	0.2120	0.0390*	0.0870	0.6148
Desmin	0.3950	0.1030	0.7144	0.2150	0.3320	0.3864
Hand2	-	-	-	0.0870	0.1760	0.3609
Cytoplasmic dynein light-intermediate chain 1 (DLIC1)	0.1120	0.3120	0.2669	0.0450*	0.0150*	0.4997
Dipeptidylpeptidase 3	0.4620	0.3870	0.9269	0.4830	0.6660	0.6671
Dullard	-	-	-	0.9970	0.2390	0.7573
Dystroglycan (DAG1)	0.0200*	0.0180*	0.4725	0.9880	0.9720	0.6865
Dystrophin	-	-	-	-	-	-
Helix-loop-helix transcription factor XE1	0.7510	0.8880	0.4700	0.4400	0.7140	0.5255
E2 (transcription factor E2)	0.0750	0.1690	0.4647	0.0810	0.0830	0.6111
met-mesencephalon-olfactory transcription factor 1 (Ebf2)	0.7320	0.1890	0.1397	0.6830	0.8520	0.2250
CCAAT/enhancer binding protein (C/EBP), alpha	0.8830	0.9490	0.8050	0.1540	0.4400	0.7463
Endothelin receptor type A	0.8160	0.8900	0.9590	0.2060	0.0520	0.3882
EF (Elongation Factor-1 alpha, 42Sp48)	-	-	-	0.6735	0.6400	0.6702
Aurora kinase A (EG2)	0.3510	0.6200	0.7224	0.1830	0.0170*	0.6086
Engrailed 2 (EN2)	0.0560	0.1510	0.0385*	0.2880	0.4300	0.1781
Enkephalin A (proenkephalin A)*	0.3460	0.1660	0.1074	0.0060*	0.0230*	0.3346
ENO (2-phosphoglycerate dehydratase, enolase)	-	-	-	0.6460	0.6550	0.0764
Era (Estrogen Receptor alpha)	0.8590	0.9560	0.8939	0.2690	0.0540	0.1565
Enhancer of split groucho	-	-	-	0.9710	0.9840	0.6392
Enhancer of zeste	0.0590	0.1220	0.6242	0.2060	0.1450	0.2269
Focal adhesion kinase	0.4990	0.6620	0.4403	0.9550	0.9960	0.8084

Transcription factor (XLFB1)	0.0065*	0.0135*	0.6450	0.0510	0.0860	0.4077
XFD-4	0.0640	0.2600	0.1903	0.0490*	0.0100*	0.1606
Flap endonuclease-1	0.4820	0.1360	0.1732	0.1800	0.1740	0.7857
FetuinB	0.2430	0.0460*	0.1642	0.0970	0.1785	0.8815
Ftz-F1-related orphan receptor (xFF1r)	-	-	-	0.2580	0.3030	0.7223
FGF (embryonic fibroblast growth factor 4)	0.0370*	0.1190	0.0161*	0.8960	0.9440	0.4253
Fibroblast growth factor receptor	0.1220	0.0390*	0.0096*	0.1170	0.2820	0.3214
Fibrinogen alpha	0.9360	0.8470	0.6547	0.0790	0.1480	0.9566
Flotillin	0.2900	0.3810	0.5994	0.1060	0.1100	0.7183
fms-related tyrosine kinase 1	0.1570	0.0860	0.1745	0.9250	0.9295	0.8388
Fms-interacting protein (NF2/meningioma region)	0.1090	0.2410	0.8749	0.4720	0.1460	0.1974
alpha-fodrin (Xen alpha 1) spectrin, non-erythrocytic 1	0.7290	0.2140	0.8795	0.2800	0.1920	0.4670
c-fos proto-oncogene	-	-	-	0.9110	0.7700	0.5726
Succinate dehydrogenase complex, Flavoprotein	0.2180	0.5340	0.4492	0.2970	0.3850	0.0920
Frequenin	-	-	-	0.5350	0.6050	0.2395
Fascin	0.9360	0.2290	0.6556	0.4370	0.7640	0.4719
Furin*	0.7300	0.8900	0.6233	0.2700	0.0210*	0.2427
Fused toes homolog	0.6440	0.9130	0.4064	0.8050	0.1930	0.2861
FYN (proto-oncogene c-fyn)	-	-	-	0.6440	0.8310	0.6285
Galectin	0.0120*	0.0240*	0.0067*	0.6710	0.7610	0.5696
alpha subunit of Gq Gtp-binding protein (G protein)	-	-	-	0.0850	0.0090*	0.0896
GATA-binding protein transcription factor GATA-1	0.6110	0.7870	0.5850	0.9690	0.7575	0.7088
Transcription factor xGata5	0.1780	0.3270	0.5439	0.8810	0.8680	0.5895
Growth hormone A	-	-	-	0.3020	0.4050	0.9991
Guanylate kinase 1	0.6450	0.5520	0.7104	0.3850	0.0610	0.1831
Glycogenin 1 (mitotic phosphoprotein 45)	0.2920	0.4810	0.0168*	0.4530	0.5320	0.0426*
Holocytochrome c synthase (heme-lyase) (hccs-prov)	0.8270	0.8880	0.6069	0.0000*	0.0030*	0.1192
cephalic Hedgehog, sonic hedgehog protein 4	0.1360	0.2550	0.7561	1.0000	0.2430	0.2291
Transcription factor XHEN1	0.5965	0.8335	0.8002	0.4720	0.3660	0.6467
Hypoxia-inducible factor 1 alpha	0.3860	0.4590	0.4948	0.2880	0.3720	0.3111
SafA - scaffold attachment factor A	0.0830	0.0400*	0.5762	0.0020*	0.0320*	0.9182
Homeobox 2/2.3*	0.2830	0.1520	0.4867	0.0430*	0.0730	0.6195
Insulin*	0.2435	0.3705	0.2870	0.7440	0.1270	0.3927
Integrin beta-1 subunit*	0.2110	0.0120*	0.1276	0.0770	0.2850	0.7590
Inversin	0.3760	0.1510	0.0062*	0.5350	0.8610	0.7457
Ubiquitin carboxyl-terminal hydrolase 5 (Isopeptidase T)	0.3420	0.0890	0.0415*	0.9170	0.3930	0.2310
Kf-1 protein (Adgr34)	0.4400	0.5920	0.4633	0.2820	0.0440*	0.3209
Kit receptor tyrosine kinase (c-kit)	0.9840	0.9720	0.3527	0.3110	0.6370	0.4923
Kinesin-like protein 2	0.3860	0.5980	0.8574	0.7350	0.2540	0.4116
L1 (ribosomal protein L1)	-	-	-	0.2470	0.5680	0.7042
L14 (ribosomal protein L14)	0.3705	0.6370	0.5735	0.8860	0.9290	0.4501
Lamin B	0.1100	0.0380*	0.3943	0.9400	0.9240	0.7441
Lamina associated polypeptide 2	0.9880	0.6750	0.1766	0.5910	0.6820	0.8860
Clathrin, light polypeptide (Lcb)	0.8260	0.9280	0.6637	0.2010	0.2860	0.0051*
Lactate dehydrogenase	0.7910	0.7760	0.4370	0.0770	0.1600	0.0327*
LEF-1 (lymphoid enhancer factor)	0.0390*	0.1130	0.7870	0.0240*	0.0650	0.8252
TGF-beta family member Lefty-A	0.0600	0.1750	0.0729	0.9070	0.1470	0.1087
LIM domain binding protein	-	-	-	0.3020	0.4630	0.1558
Lipocalin (Ptgds)	0.1710	0.2980	0.4336	0.4170	0.3150	0.0834
Lpa1R (lysophosphatidic acid receptor)	-	-	-	0.3450	0.0910	0.0093*
LR (Leptin Receptor)	-	-	-	0.7390	0.5160	0.1189

Lipoprotein (LDL) receptor-related protein 6	-	-	-	0.9160	0.1930	0.6215
Autoantigen La (La protein)	0.2540	0.0880	0.3856	0.2680	0.3840	0.8943
Microfibrillar-associated protein 1	0.7820	0.9060	0.9091	0.3995	0.6050	0.8731
Myristoylated alanine-rich C kinase substrate	0.0430*	0.1350	0.4264	0.9825	0.9830	0.4463
XMax2 and XMax4	-	-	-	0.0955	0.1825	0.4937
Myogenin	0.7540	0.8170	0.9276	0.0150*	0.0470*	0.1267
Myozenin1	0.9160	0.4210	0.7304	0.4280	0.3700	0.4516
N-CAM (neural cell adhesion molecule)*	0.3210	0.0610	0.2543	0.8070	0.2460	0.1001
NF-M1 (middle molecular neurofilament)	0.7300	0.6870	0.7659	0.1270	0.3350	0.7672
neurogenin-related 1 (X-NGNR-1)	0.8650	0.9730	0.9218	0.3230	0.3000	0.7386
Internexin neuronal intermediate filament protein	0.0510	0.0160*	0.0053*	0.7430	0.7570	0.2816
NK3 transcription factor related, koza	0.4280	0.6400	0.0406*	0.0370*	0.0590	0.0399*
Nonmuscle myosin II heavy chain A	0.9980	0.9470	0.3710	0.1110	0.0800	0.2255
Nonmuscle myosin heavy chain B	-	-	-	0.0930	0.0580	0.9894
Nucleolar-localized protein NO38	0.9130	0.9320	0.7492	0.8350	0.7310	0.3799
Nucleobindin 1	-	-	-	0.4760	0.6070	0.4252
Nucleoplasmin	0.9280	0.8990	0.9963	0.9600	0.9230	0.8325
Nucleoporin (Nup88)	0.7300	0.8010	0.4510	0.2570	0.5520	0.2907
OLPA (Dorphin)	0.4560	0.6580	0.1583	0.9470	0.9780	0.5285
Olfactory marker protein (XOMP)	0.1330	0.2650	0.5306	0.5180	0.7460	0.0215*
OncogenesC-ets-1 (c-ets-1 proto-oncogene)*	0.4370	0.5880	0.8562	0.3230	0.4850	0.7489
OncogenesC-ets-2 (c-ets-2 proto-oncogene)*	0.1075	0.3050	0.2922	0.9440	0.6940	0.9549
OncogenesC-myc (myelocytomatosis)*	0.8950	0.8600	0.6704	0.8700	0.3820	0.6145
Dynactin 2 (p50)	0.7620	0.8890	0.4371	0.6740	0.1360	0.1604
PACSIN2	0.6450	0.5570	0.4387	0.1490	0.0300*	0.7350
Convertase PC2	0.9310	0.9200	0.8691	0.9770	0.7990	1.0000
Prolyl isomerase (Pin1)	0.9520	0.9340	0.4994	0.8320	0.9180	0.9834
PKC (protein kinase C,delta)	0.8900	0.9675	0.3247	0.5820	0.7520	0.7905
Plakoglobin	0.9010	0.9790	0.8255	0.9120	0.9540	0.9874
Peripheral myelin protein 22	-	-	-	0.0490*	0.0170*	0.0531
POMC (pro-opiomelanocortin)*	0.1680	0.2990	0.9937	0.0590	0.1780	0.5733
POU domain Gene 1	-	-	-	0.6580	0.8700	0.5370
POU3	-	-	-	0.0730	0.1500	0.5944
Phosphorylase phosphatase (Ppp2B)	-	-	-	0.4770	0.0750	0.8485
Protein phosphatase 4, regulatory subunit 2 (Ppp4r2)	0.3360	0.4300	0.3293	0.8360	0.8900	0.1810
LIM protein Prickle	0.5210	0.5850	0.2356	0.6660	0.7960	0.1445
Prolactin Receptor	0.8950	0.9400	0.5260	0.3670	0.6460	0.0834
Prothymosin	0.6825	0.7520	0.9170	0.9450	0.2720	0.1542
Phosphorylase, glycogen; brain	0.7800	0.2840	0.8804	0.0720	0.2070	0.5815
RAB18 (member RAS oncogene family)	-	-	-	0.1870	0.3570	0.5102
Rac GTPase	-	-	-	0.4420	0.4800	0.4481
Rad51	-	-	-	0.0780	0.2670	0.0483*
Rag-1	0.4860	0.7290	0.4375	0.1530	0.3000	0.0195*
Ral interacting protein (rlip) - RalA (RalB-binding protein)	0.6390	0.7060	0.9260	0.3500	0.6870	0.6030
RalB	-	-	-	0.4960	0.7190	0.8398
Retinoic acid receptor alpha	0.8690	0.9370	0.0443*	0.1050	0.0170*	0.5800
RDS35 (retinal degradation slow/peripherin)	0.1450	0.0480*	0.0889	0.8570	0.9360	0.5515
RDS38/peripherin	0.4350	0.6150	0.2368	0.2150	0.0060*	0.5074
Requiem	0.8220	0.8800	0.9277	0.8580	0.7810	0.1581
Rhodopsin	-	-	-	0.7550	0.1360	0.8260
Ringo (p33 ringo, ls26)	0.4890	0.6930	0.3147	0.9060	0.8100	0.2189

RIO kinase 2	0.0040*	0.0080*	0.1706	0.1570	0.3850	0.7642
Rwdd1 (RWD domain containing 1)	0.9650	0.9300	0.6886	0.9830	0.3590	0.5107
Retinal homeobox A	0.6920	0.7470	0.8166	0.1990	0.4380	0.9749
Rxrb (retinoid X receptor beta)	0.0690	0.1790	0.1411	0.6290	0.8350	0.9051
Sister chromatid cohesion establishment factor (SCC2)	0.1740	0.3000	0.1234	0.8160	0.7580	0.9521
Syndecan 2 (heparan sulfate proteoglycan 1)	0.0560	0.1240	0.6430	0.6160	0.7800	0.0112*
Sek-1 receptor tyrosine kinase	0.4450	0.2700	0.7852	0.6240	0.7470	0.5719
Selenoprotein I	0.9560	0.3960	0.6264	0.0890	0.1000	0.0917
Selenoprotein T	0.5485	0.4950	0.6713	0.5470	0.5950	0.7394
Septin 11	0.7220	0.8160	0.4990	0.8600	0.8290	0.7254
Septin A (XlSeptA)	0.4365	0.6625	0.8783	0.3250	0.2040	0.1425
serum/glucocorticoid regulated kinase	-	-	-	0.7270	0.6350	0.8501
Shab12	0.5655	0.6585	0.7982	0.2110	0.4150	0.5980
Siah-interacting protein	0.7560	0.9400	0.7691	0.8070	0.8500	0.9484
Sloan-Kettering viral oncogene homolog	0.2695	0.5020	0.9736	0.5360	0.6530	0.3125
Histone stem-loop binding protein (SLBP)	0.6610	0.6700	0.5797	0.0030*	0.0020*	0.8836
Suc1-associated neurotrophic factor target	0.9550	0.6790	0.3862	0.7130	0.8240	0.6105
Sox11 (XLS13)	0.9730	0.7600	0.8218	0.9280	0.8150	0.6637
Sox17a (HMG box transcription factor Sox17-alpha)	0.9110	0.9370	0.6604	0.1985	0.3790	0.0410*
Sox18 (Transcription factor SOX-18)	0.3730	0.3430	0.8661	0.1230	0.2990	0.0399*
SP22	-	-	-	0.8350	0.5630	0.3881
Sparc	0.7860	0.2890	0.1551	0.8310	0.9340	0.7497
Spats2 (spermatogenesis associated, serine-rich 2)	0.2910	0.2240	0.1836	0.2930	0.4000	0.5665
Spermatid perinuclear RNA binding protein	0.2420	0.1660	0.2982	0.8430	0.9310	0.7468
Sprouty-2	0.0490*	0.1910	0.1259	0.5870	0.7770	0.3808
Sulfide quinone reductase-like	0.0730	0.1980	0.8676	0.2700	0.2460	0.0742
Src (pp60c-src protein)	0.2630	0.4510	0.6295	0.4295	0.7140	0.5936
Stanniocalcin 1	0.5290	0.3150	0.2126	0.6770	0.7030	0.9394
Staufen 1	0.6000	0.8130	0.2474	0.2915	0.4800	0.8918
Stress-induced-phosphoprotein 1	0.4100	0.5790	0.6479	0.0090*	0.0680	0.6135
Stomatin	-	-	-	0.8120	0.3930	0.8533
Strabismus	-	-	-	0.7430	0.8270	0.6505
SUG1	-	-	-	0.8220	0.8380	0.7509
translation initiation factor SUI1	-	-	-	0.2230	0.4310	0.3848
Sumo	-	-	-	0.7760	0.6540	0.8864
Survivin (Xsvv1)	0.2610	0.3770	0.1277	0.0610	0.0760	0.0150*
Synaptobrevin	-	-	-	0.0190*	0.0550	0.0049*
Synaptophysin	0.0700	0.1200	0.4390	0.0220*	0.0050*	0.6087
Xwnt8 inhibitor sizzled (szl) (putative wnt inhibitor frzb3)	0.5600	0.2660	0.1614	0.7100	0.8370	0.6371
TAF-Ibeta	-	-	-	0.2660	0.4250	0.1651
T-box transcription factor Tbx5	-	-	-	0.0900	0.1310	0.6234
TCRzeta subunit	0.6870	0.1720	0.8223	0.1470	0.2230	0.8226
Bax Inhibitor-1, testis enhanced gene transcript	0.9190	0.4110	0.0835	0.8650	0.9540	0.5544
TRK-fused protein TFG	0.9500	0.9570	0.7025	0.8050	0.9040	0.7929
Thyroid Hormone Receptor alpha*	-	-	-	0.2570	0.3870	0.0537
Thyroid Hormone Receptor beta*	0.0170*	0.1330	0.4844	0.9530	0.7670	0.7169
Mesoderm Posterior (Mesp)	0.4630	0.3260	0.9666	0.8170	0.7180	0.4615
cytotoxic granule-associated RNA binding protein (TIA1)	0.2810	0.0570	0.1501	0.0990	0.2030	0.1358
TIAR	0.5270	0.6340	0.3010	0.9210	0.7800	0.2212
Tyrosine kinase	0.4330	0.6350	0.1190	0.3170	0.5000	0.5804
IGF (Insulin-like Growth Factor) Receptor	-	-	-	0.1030	0.1840	0.1212

Transducer of erbB	-	-	-	0.8875	0.7240	0.6015
Transferrin	0.4500	0.2500	0.5726	0.0370*	0.0290*	0.7927
Thyrotropin-releasing Hormone	0.9500	0.5180	0.2479	0.7560	0.8420	0.3405
Thyrotropin-releasing Hormone Receptor 1	0.5320	0.0480*	0.7070	0.9910	0.9970	0.1205
Neurotrophin receptor B xTrkB-alpha	0.9490	0.9470	0.7177	0.2280	0.1330	0.0964
fast skeletal Troponin C	-	-	-	0.0770	0.2090	0.5216
unitary non-NMDA glutamate receptor subunit U1	0.7040	0.8490	0.2989	0.9430	0.4640	0.3726
Ubiquiton-conjugating enzyme e2e	0.4910	0.8440	0.4993	0.4000	0.3490	0.4857
xUBF mRNA for upstream binding factor	0.7980	0.8860	0.2699	0.8510	0.3910	0.8087
endoplasmic reticulum UDP-Glc/UDP-Gal transporter	-	-	-	0.0960	0.0690	0.7941
UDP-glucose ceramide glucosyltransferase	0.7925	0.8770	0.9496	0.6770	0.6770	0.4455
Uroplakin 1A	0.4600	0.5840	0.0444*	0.0720	0.0870	0.5115
Ubiquinol-cytochrome C reductase complex	0.6230	0.0700	0.1405	0.3330	0.1720	0.6988
Vasodilator-stimulated phosphoprotein	0.5510	0.6800	0.3386	0.1890	0.3460	0.1994
Ventral anterior homeobox protein (Vax1)	0.8400	0.3190	0.7649	0.8860	0.3990	0.8373
Ventral anterior homeobox protein (Vax2+3)	0.6090	0.5930	0.1202	0.7720	0.8220	0.7571
Von Hippel-Lindau binding protein 1	0.1505	0.1650	0.3194	0.7070	0.7910	0.5620
Vg1 RNA binding protein	0.5360	0.6320	0.8041	0.8670	0.5650	0.3260
Vimentin*	0.1470	0.3280	0.3921	0.7860	0.7540	0.5623
Tryptophanyl-tRNA synthetase	0.7810	0.3300	0.5103	0.6000	0.6890	0.0039*
Wee1A kinase	0.0970	0.2020	0.0660	0.2340	0.5120	0.9277
Wee1B, Wee1-like protein kinase	0.9840	0.7280	0.7931	0.7710	0.9380	0.1694
Uterine sensitization-associated protein-1 (Wise-A)	0.0420*	0.0810	0.1216	0.1900	0.1670	0.1914
Xwnt-3	-	-	-	0.3185	0.3690	0.4151
Wilms' tumor suppressor (WT1)	0.7780	0.8320	0.7098	0.1450	0.4540	0.3598
Cofilin (XAC)	0.1640	0.2510	0.9734	0.8820	0.8890	0.6422
XE2 (helix-loop-helix transcription factor E2)	-	-	-	0.7920	0.7570	0.2385
Xefiltin	0.4580	0.1710	0.0494*	0.0220*	0.0270*	0.0595
Epidermis specific serine protease Prss27 (Xepsin)	0.9450	0.2570	0.1439	0.9700	0.9770	0.4597
Fork head related (XFD1)	0.2590	0.3860	0.0902	0.9960	0.7630	0.5750
Fork head protein (XFD2)	0.3555	0.0660	0.7029	0.9400	0.9670	0.8595
Interleukin-1 beta-converting enzyme (Caspase 1)	0.1570	0.1240	0.8462	0.1510	0.3260	0.5056
Xlmb (maternal B9.10 and B9.15 protein)	0.2820	0.3970	0.0270*	0.1200	0.1450	0.1260
L-myc oncogene (xL-myc)	0.2650	0.1680	0.1837	0.0250*	0.0070*	0.1856
Xnot (homeobox protein)	0.2560	0.1340	0.8942	0.0950	0.1770	0.5087
TGF-beta related growth factor Xnr-4 (Xnr4)	0.5660	0.0350*	0.2998	0.0730	0.1270	0.9123
XrnF12	0.1590	0.1970	0.7625	0.9740	0.9970	0.6248
Xrpf (XrpFI beta 1) GA binding protein transcription factor	0.1800	0.2550	0.0086*	0.1360	0.1890	0.3775
ZFTF (zinc finger transcription factor SLUG)	-	-	-	0.7740	0.2720	0.5955
ZPB (zona pellucida glycoprotein)	0.9910	0.6300	0.3384	0.7100	0.8575	0.6232
